# MicroRNA regulation and its effects on cellular transcriptome in Human Immunodeficiency Virus-1 (HIV-1) infected individuals with distinct viral load and CD4 cell counts

**DOI:** 10.1186/1471-2334-13-250

**Published:** 2013-05-30

**Authors:** Karolina Duskova, Pruthvi Nagilla, Hai-Son Le, Priyadarshini Iyer, Anbupalam Thalamuthu, Jeremy Martinson, Ziv Bar-Joseph, William Buchanan, Charles Rinaldo, Velpandi Ayyavoo

**Affiliations:** 1 Department of Infectious Diseases and Microbiology, Graduate School of Public Health, University of Pittsburgh, 425 Parran Hall, 130 Desoto Street, Pittsburgh, PA, 15261, USA; 2 Department of Machine Learning, Carnegie Mellon University, Pittsburgh, PA, USA; 3 Human Genetics, Genome Institute of Singapore, 60 Biopolis Street 02-01, Singapore, Singapore

## Abstract

**Background:**

Disease progression in the absence of therapy varies significantly in HIV-1 infected individuals. Both viral and host cellular molecules are implicated; however, the exact role of these factors and/or the mechanism involved remains elusive. To understand how microRNAs (miRNAs), which are regulators of transcription and translation, influence host cellular gene expression (mRNA) during HIV-1 infection, we performed a comparative miRNA and mRNA microarray analysis using PBMCs obtained from infected individuals with distinct viral load and CD4 counts.

**Methods:**

RNA isolated from PBMCs obtained from HIV-1 seronegative and HIV-1 positive individuals with distinct viral load and CD4 counts were assessed for miRNA and mRNA profile. Selected miRNA and mRNA transcripts were validated using in vivo and in vitro infection model.

**Results:**

Our results indicate that HIV-1 positive individuals with high viral load (HVL) showed a dysregulation of 191 miRNAs and 309 mRNA transcripts compared to the uninfected age and sex matched controls. The miRNAs miR-19b, 146a, 615-3p, 382, 34a, 144 and 155, that are known to target innate and inflammatory factors, were significantly upregulated in PBMCs with high viral load, as were the inflammatory molecules CXCL5, CCL2, IL6 and IL8, whereas defensin, CD4, ALDH1, and Neurogranin (NRGN) were significantly downregulated. Using the transcriptome profile and predicted target genes, we constructed the regulatory networks of miRNA-mRNA pairs that were differentially expressed between control, LVL and HVL subjects. The regulatory network revealed an inverse correlation of several miRNA-mRNA pair expression patterns, suggesting HIV-1 mediated transcriptional regulation is in part likely through miRNA regulation.

**Conclusions:**

Results from our studies indicate that gene expression is significantly altered in PBMCs in response to virus replication. It is interesting to note that the infected individuals with low or undetectable viral load exhibit a gene expression profile very similar to control or uninfected subjects. Importantly, we identified several new mRNA targets (Defensin, Neurogranin, AIF) as well as the miRNAs that could be involved in regulating their expression through the miRNA-mRNA interaction.

## Background

HIV-1 infected individuals show remarkable variation in virus replication and disease progression [1,2]. Recent studies support the notion that the host cellular gene expression profile (the transcriptome), in the context of virus infection, is directly correlated with disease patterns [3-5]. The gene expression pattern associated with HIV-1 infection in cells is likely controlled by host genetics and external factors, leading to dysregulated antiviral activity, inflammatory response and disease progression. The replication, spread, and immune evasion of the virus and the progression of disease depend on host cellular transcription and gene regulation in virus-specific target cells and bystander cells [6-8]. As virus replication is dependent on host cellular machinery, high viral load (HVL) augments cell destruction and gene regulation, whereas low viral load (LVL) or undetectable viral load promotes latency and possibly immune control. Further, increased virus production also results in release of free extracellular (cell- and virus-free), virion-associated, and cell-associated viral antigens, as well as infectious and noninfectious virus particles that could potentially alter bystander cell transcriptome and destruction. Consistent with this scenario, HIV-1 virus with defective expression of viral proteins such as Nef, Vpr, Gag, and Pol is shown to induce differential gene expression [9-11].

Host factors bearing on the outcome of HIV-1 infection include genetic elements such as HLA alleles, polymorphisms in HIV-1 receptors and coreceptors, and genes involved in innate and adaptive immune responses [12-15]. Following the landmark discovery of the *CCR5*-Δ32 mutation that protects against HIV-1 infection [16-18], many other genetic variants have been shown to affect HIV-1 infection and AIDS pathogenesis [12,19,20]. It is likely that, in addition to incomplete immunological control, host genetic variation and differences in gene expression in the infected host cells may also contribute to the differential disease pattern [21-23]. Along these lines, attempts were made to identify host cellular proteins associated with HIV-1 infection [24-27]. There is limited information, however, regarding the regulation of host cellular genes at the transcriptional, post-transcriptional, and translational levels.

Previous studies have shown that HIV-1 infection differentially regulates host cellular factors responsible for inflammation, immune response, cell cycle/proliferation and apoptosis, suggesting that differential gene expression in infected individuals either accelerates disease progression or enhances resistance to the development of disease [28-30]. Recent discoveries have emphasized a central role for the new class of small non-coding RNA in gene expression controlling growth, development, and immune response *in vivo*[31-33]. Regulation of gene expression by miRNA occurs primarily at the post-transcriptional level [34,35]. Recent studies have shown that miRNA have a unique expression profile in cells of the innate and adaptive immune systems, CNS, and cancers [36-39]. Based on these observations, we hypothesize that pathogens including viruses could potentially modulate host cellular transcription at multiple levels by targeting various factors including miRNAs.

Studies previously have evaluated the expression of either miRNA or mRNA using PBMCs, CD4+ T-lymphocytes, CD8+ T-lymphocytes, monocytes and/or brain tissue from HIV-1 infected subjects [4,40-48]. Results from these studies have identified a number of cell specific miRNAs that are differentially regulated by HIV-1 infection. Similarly regulated mRNA transcripts targeting IFN, cytokine-cytokine receptor signaling, apoptosis and MAPK signaling were also regulated by HIV-1 infection. However, in the case of HIV-1 infection, few combined miRNA and mRNA analyses using RNA isolated from the same sample or at the same time point have been conducted, except for the recent studies by Zhou et al [49] and Chang et al [50], using HIV-1 subjects brain tissue and infected CD4+ T cells, respectively. These studies provided the first line of evidence for a functional relationship between miRNA and mRNA in the context of HIV-1. In an effort to understand miRNA-mRNA interactions in the host during HIV-1 infection, we performed comparative global miRNA and mRNA microarray profiling in PBMCs derived from HIV-1 infected individuals with high and low or undetectable viral load and compared these with age and sex matched uninfected controls. Our results indicate that individuals with high viral load showed significant differences in both their miRNA and mRNA profiles compared to subjects with low viral load. In HIV-1 positive subjects with >45,000 copies/ml of viral RNA, a high proportion of miRNAs (178/191) showed increased expression (with >2-fold change, p value of <0.05), whereas the mRNA profile showed opposing results with a higher number of genes (184/308) being downregulated. The regulatory network analysis revealed that several host cellular factors, implicated in HIV-1 disease progression, might be modulated through miRNA regulation of gene expression during disease development. Taken together, these results provide evidence that the miRNA profile could be an early indicator of HIV-1 induced host cellular dysfunction.

## Results

### miRNA profiling in control versus HIV-1 positive groups

RNA isolated from HIV-1 subjects with high and low viral loads, and from control subjects were subjected to miRNA profiling and data analyses to identify the differentially expressed miRNA within these groups. RNA isolated from PBMCs was tested for RNA quality and integrity by Bioanalyzer and by Taqman assays for endogenous control miRNAs and mRNA species. Specifically, the samples were tested for RNU48, miR-26b, or U6, and assessed by the absolute Ct value of <25. The expression profile of 754 host cellular miRNAs (excluding endogenous controls) was assessed in PBMCs obtained from HIV-1 negative and HIV-1 positive subjects. Results from high and low viral load groups were compared with the uninfected control group as well as within the HIV-1 positive groups. Differentially regulated miRNAs between these groups were analyzed using two different software packages with the appropriate settings required for each software to maximize the confidence. The first program used was DataAssist with default settings (maximum allowable Ct value was 40), as suggested by the manufacturer. The Benjamini-Hochberg False Discovery Rate adjustment was applied for multiple comparison corrections. Our results indicate that 38, 244 and 183 miRNAs were differentially regulated in control versus LVL, control versus HVL, and LVL versus HVL respectively, with at least a 2-fold change and p value <0.05 (Table 1).

**Table 1:** Differentially regulated miRNA in low and high viral load groups compared to uninfected control group using two different software with statistical significance

	**Differentially regulated miRNA+/- 2-fold p < 0.05**			**Differentially regulated miRNA+/- 2-fold p < 0.01**		
	**DataAssist**	**StatMiner**	**Common**	**DataAssist**	**StatMiner**	**Common**
Control vs. LVL	38	41	21	8	14	8
Control vs. HVL	244	218	191	163	137	118
LVL vs. HVL	183	182	158	90	99	80

Comparative analysis of significantly differentially regulated miRNAs between Statminer (Integromics) and DataAssist (Applied Biosystems). For both analyses, RNU44, RNU48, and ath-miR-159 controls were eliminated from the analysis. For DataAssist, all miRNAs with CT valve of <40 were considered.

For the StatMiner analysis, miRNAs that show a raw Ct value of <36, and miRNAs that are expressed in >25% of the subjects in each group, were included as suggested [51]. This resulted in the elimination of 177, 173 and 160 miRNAs from each comparison (control vs LVL; Control vs HVL; LVL vs HVL) respectively. The remaining miRNA were assessed using a statistical package to identify the significantly regulated miRNA within and between these groups. Our results indicate that HIV-1 infection differentially regulated expression of several miRNAs (+/- 1.72-fold with p < 0.05 using the Benjamini-Hochberg method within each group (Additional file 1: Table S1)). The level of expression in infected subjects was compared with that in uninfected controls, and fold differences were calculated based on normalization with endogenous control, U6 snRNA/mammU6, as suggested by the manufacturer. Our results indicate that 41, 218 and 182 miRNAs were differentially regulated with +/- 2-fold change and p < 0.05 in control versus LVL, control versus HVL and LVL versus HVL, respectively (Table 1). To further increase the confidence, miRNAs that were identified as differentially regulated by both programs were assessed, and 21, 191 and 158 of miRNAs were commonly found in both analyses (p < 0.05) of control versus LVL, control versus HVL and LVL versus HVL, respectively. However, as the significance increases to p < 0.01 both analyses identified more than 70% of miRNAs commonly.

### Differential regulation of miRNA in HIV-1 subjects with a broad range of viral load and CD4 counts

Among the 754 miRNAs tested, 21 miRNAs were differentially regulated in low viral load individuals compared to uninfected controls using two different softwares (Table 1). Of these, 2 miRNAs were downregulated and 19 miRNAs were upregulated (fold change ranging from 3.09 to 20.48). However, in the HVL group, the number of differentially regulated miRNAs was significantly higher. A total of 191 miRNAs were differentially regulated in HVL subjects compared to uninfected controls. Of these, 13 were downregulated (fold change ranging from -2.05 to -89.82) and 178 were upregulated. Similarly, we identified 158 miRNAs that were differentially regulated when we compared the LVL and HVL groups. Of these, 27 miRNAs were downregulated and 131 miRNAs were upregulated, with the fold change ranging from 1.73 to 6460.41. Together these results indicate that HIV-1 infection upregulated expression of several miRNAs and the effect was more significant in high viral load group compared to low viral load group.

**Figure 1:**
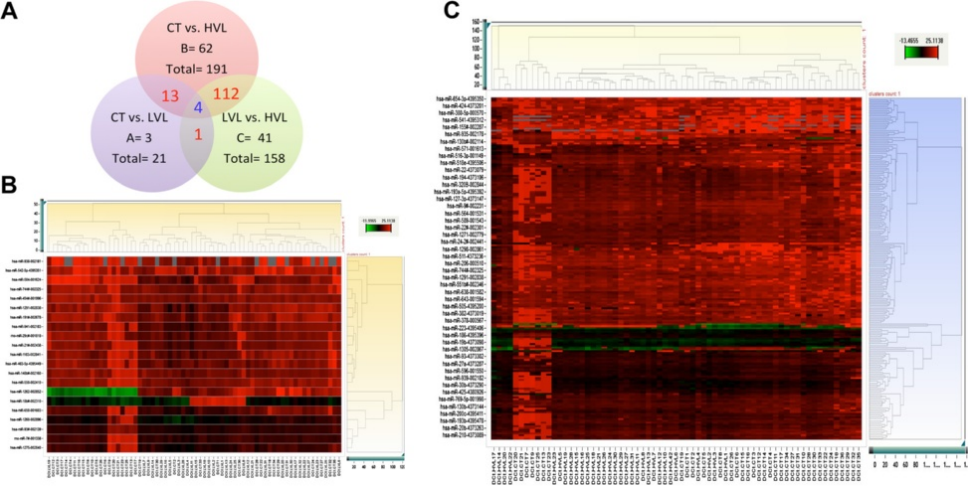
**A: Expression profile of differentially regulated miRNAs in HIV-1 infected versus uninfected subjects.** (**A**) The Venn diagram displays the number and overlap of significantly differentially expressed miRNA (Benjamini-Hochberg adjusted, p < 0.05) among the LVL and HVL groups relative to the CT and within the infected groups. Hierarchical clustering of miRNA between CT and LVL (**B**); and CT and HVL (**C**). MicroRNA in the clustergram are dysregulated at a significance cutoff of p < 0.05. The dendogram depicting the clustering of samples is calculated using Complete linkage with Euclidian distance measure values. Color ranging from green to red indicates minimum to maximum ∆CT. Numbers on X-axis represent subject group. CT, uninfected controls; LVL, low viral load subjects; HVL, high viral load subjects. The clustergrams were generated using StatMiner analyses.

Figure 1A shows the miRNAs that are specific to each group, as well as the overlap between the groups. When uninfected controls (CT) are compared with HIV-1 infected samples, 17 miRNAs are commonly dysregulated in the HIV-1 infected subjects (LVL and HVL combined). Among the 21 miRNAs that are differentially regulated between the CT vs. LVL groups, 3 were unique to the LVL group, whereas when the CT and HVL groups are compared, 62 of the 191 differentially regulated miRNAs were specific to the HVL group. Similarly, we also compared all three groups and found that 4 miRNAs are commonly dysregulated in all three groups. Further examination indicates that miR-1275, miR-483-5p, and miR-650 show similar upregulation in the HVL group compared to either the CT or LVL group, whereas miR-1262 shows downregulation in the CT versus HIV-1 positive group, and was upregulated in the HVL group compared to the LVL group. Further analyses within the HVL group based on viral load ranging from 40,000 to 600,000 RNA copies/ml did not show any additive effect. Together, these results suggest that HIV-1 infection has specific regulatory effects on miRNA expression profiles.

To further assess the clustering of subjects within these groups and across the groups, hierarchical clustering was performed in a comparison of the control and LVL groups (Figure 1B) and of the control and HVL groups (Figure 1C). When the control and LVL groups are compared, subjects from each group formed several clusters and these clusters dispersed intermittently. Many of the control samples formed a single major cluster, while the remaining control samples were interspersed among the LVL subjects in two other major clusters. However, when we compared the control and HVL groups (Figure 1C), the majority of the subjects from each group clustered distinctly from each other based on their miRNA profile, with an exception of a single separate cluster of HVL containing 4 subjects and control group with 6 subjects from each group. Further analyses of CD4 counts, age and viral load did not show any differences compared to subjects in the major cluster. The comparison of HVL to LVL produced 3 major clusters in LVL and 2 clusters in HVL (Additional file 2: Figure S1). Importantly, these results suggest that the miRNA expression profile within the infected subjects with high viral load is specific. This pattern is different in HIV-1 subjects with undetectable viral load and high CD4 counts, who maintain an expression profile that is similar to uninfected controls. More importantly, this distinct profile is observed in most of the subjects (based on the clustering) and further confirms the specificity.

### Validation of miRNA expression by qRT-PCR

**Figure 2:**
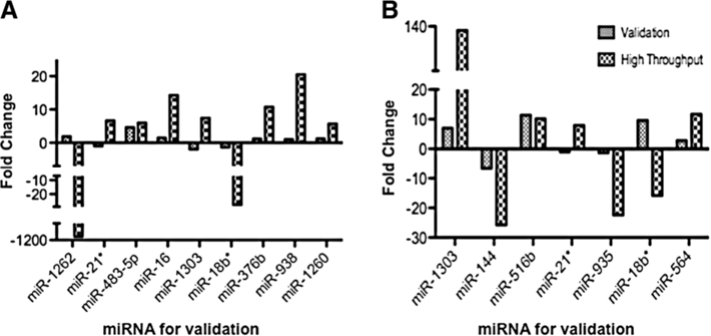
**Independent individual validations of randomly selected differentially regulated miRNAs from Taqman array platform.** qRT-PCR was used to validate the expression of selected miRNAs from the high throughput results (derived by StatMiner) using a specific primer and probe for each miRNA. (**A**) miRNAs selected from Control versus LVL comparison; (**B**) miRNAs selected from control versus HVL comparison. Fold increase/decrease was calculated based on normalization to U6. Average fold change for each miRNA represents fold change obtained from independent donors (N = 5 per group).

To validate the differentially regulated miRNAs from the microarray results, we randomly selected miRNAs (miRNAs with >2-fold change and p < 0.05) and tested them in independent subjects (using similar selection criteria) by qRT-PCR using miRNA specific Taqman primers and probes (Figure 2A&B). Results from the comparison of the LVL group and uninfected controls indicate that among the miRNAs tested, six miRNAs (miR-483-5p, miR-16, miR-18b*, miR-376b, miR-938, and miR-1260) exhibit similar fold change pattern as seen in the high throughput results. However, three miRNAs (miR-21*, miR-1262 and miR-1303) showed an opposite fold change pattern to that of the high throughput results. Specifically, miR-1262 was downregulated and miR-21* and miR-1303 were upregulated in high throughput, while in the qRT PCR, they were upregulated and downregulated, respectively (Figure 2A). Overall these results indicate about 60% validation (6/9) in terms of similar state of expression (up or down) in both the high throughput array and qRT-PCR, however the exact fold change level is different between the two systems as expected.

Similarly, we also tested randomly selected miRNAs in a set of independent HVL subjects relative to uninfected controls (Figure 2B). This comparison indicated that among the seven miRNAs tested, miR-1303, miR-516b and miR-564 showed upregulation in both high throughput array and qRT-PCR, whereas miR-144 and miR-935 showed downregulation in both the array and qRT-PCR assay (Figure 2B). However, two miRNAs (miR-21* and miR-18b*) showed no correlation between the two assays. Further analysis indicates that both miR-21* and miR-18b are low abundant miRNAs and that could be one of the reasons for the observed discrepancies. Overall, validation of miRNA in both sample groups confirms >65-85% of the miRNAs suggesting that the array results are reproducible in independent subjects and this effect is more pronounced in the HVL group compared to LVL group. These results are in accordance with the published studies [52-54], indicating that there are differences in differential regulation observed in array versus qRT-PCR assay and 100% validation is rare if the validation samples are randomly selected as done here. This is further supported when we assessed the significance level (p values) and the fold change for these miRNAs (in Additional file 3: Table S2). For instance miR-1303 is upregulated in both LVL and HVL subjects in the array, however the significance is p = 0.024 in LVL subjects and p = 4.67E-13 in HVL subjects. Validation results for miR-1303 by qRT-PCR show similar trend in HVL and opposite pattern in LVL subjects. Additionally, we also observed that five miRNAs that are in concordance with qRT-PCR have highly significant p values (<1.35E-05), whereas that of the miRNAs tested in LVL are <0.05 – 0.005. Together these results suggest that differentially regulated miRNAs with significant p values (<0.00001) has higher validation concordance rate than miRNAs selected based on higher fold change.

### HIV-1 induced gene regulation of cellular mRNA transcripts

MicroRNAs regulate cellular gene expression at the post-transcriptional level, thus silencing and/or downregulating gene expression [55,56]. To assess whether a direct correlation exists between the expression patterns of miRNA and mRNA, we assessed the expression profile of mRNA transcripts in PBMCs of the same subjects. Microarray profiling of mRNA samples was normalized with the internal endogenous control and cross-compared between the two groups. Among the 47,000 transcripts tested, 21,852 probes were detected in all samples. Among the total detected probes, 47, 11,510 and 10,007 probes were significantly (p < 0.01) regulated but less than +/- 2-fold change in control versus LVL, control versus HVL and LVL versus HVL groups, respectively. However, when we narrowed the probes with a fold change of +/- 2- (p < 0.01), we obtained 0, 309 and 182 probes in the 3 groups, respectively (Table 2).

**Table 2:** Differentially regulated mRNA probes in low and high viral load groups compared to uninfected control group with statistical significance

	**Detected mRNA probes**	**p ≤0.01 (All probes)**	**p ≤0.01 (Known probes)**	**+/- 2 fold and p ≤ 0.01 (All probes)**	**+/- 2 fold and p ≤ 0.01 (Known probes)**
Control Vs. LVL	21,852	47	41	0	0
Control Vs. HVL		11,510	9,137	309	280
LVL Vs. HVL		10,007	7,820	182	166

Differentially regulated mRNA probes in within the three study groups based on Illumina HT12 microarray data analyzed with IBD. All probes represent annotated, non-annotated, and curated probes. Known probes represent annotated probes or named genes.

**Figure 3:**
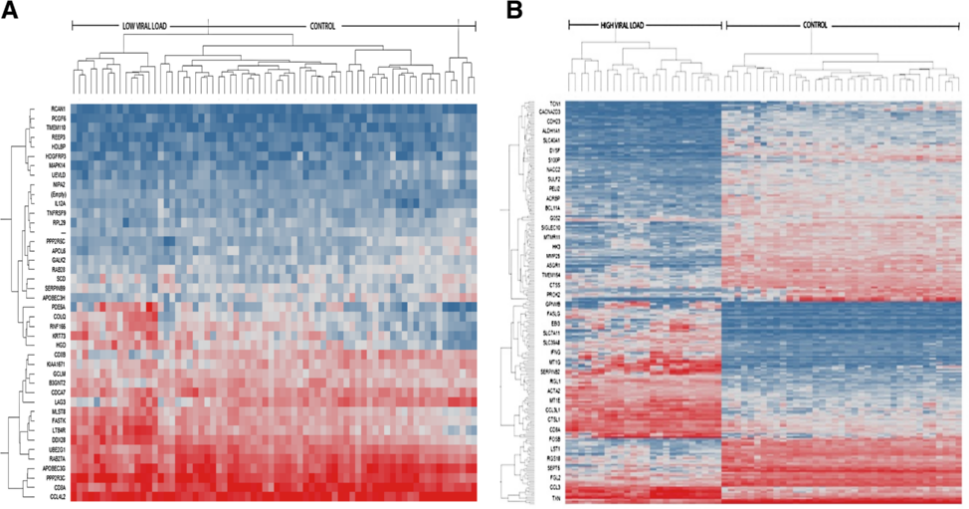
**Expression profile of differentially regulated mRNA transcripts in HIV-1 infected versus uninfected subjects.** (**A**). Hierarchical clustering of differentially regulated mRNA in LVL compared to control; and (**B**), CT and HVL. The probes in this clustergram are significantly differentially regulated (p < 0.05). Red indicates high, blue indicates low, and gray stands for no change in level of expression. Numbers on X-axis represent subject group; Y-axis represents the gene symbol. CT, uninfected controls; LVL, low viral load subjects; HVL, high viral load subjects.

Among the 47 probes (41 annotated) differently expressed in the LVL samples compared to CT (FDR corrected with p-value of <0.01), 36 were upregulated and 11 were downregulated (Table 2). None of the transcripts show more than 1 fold difference (p < 0.01) compared to controls, suggesting that there is not a greater difference between uninfected controls and infected subjects with an undetectable viral load. In contrast, 309 probes were differentially regulated in the high viral load samples compared to the uninfected controls (FDR corrected with a p-value of <0.01 with +/- 2-fold regulation). Of these, 125 were upregulated, and 184 were downregulated. Similarly, we also compared the LVL and HVL groups and identified 182 probes that are differentially regulated. Within these transcripts, a majority (2/3) of them (113) are downregulated and 69 were upregulated. Further, 178 probes were commonly shared between control versus HVL and LVL versus HVL, whereas none of the mRNAs are shared by all the groups or between control versus LVL group. More importantly, the remaining 131 probes are very specific to the HVL group. Together, these results suggest that subjects with high viral load or AIDS exhibit a unique transcriptome profile compared to the low viral load group. Similar findings were observed in other studies [4,42,44,45]. Clustergram analysis of differentially regulated mRNA profiles between control and LVL did not show distinct clustering (Figure 3A), whereas the control versus HVL group comparison showed clusters segregating distinctly, indicating a different transcriptome profile mediated by virus replication within this group (Figure 3B). Similar comparison between LVL and HVL also exhibited a distinct clustering pattern (Additional file 4: Figure S2) suggesting that virus replication has significant impact on host cellular gene expression.

### Host cellular factors, pathways and biological processes altered by HIV-1

**Figure 4:**
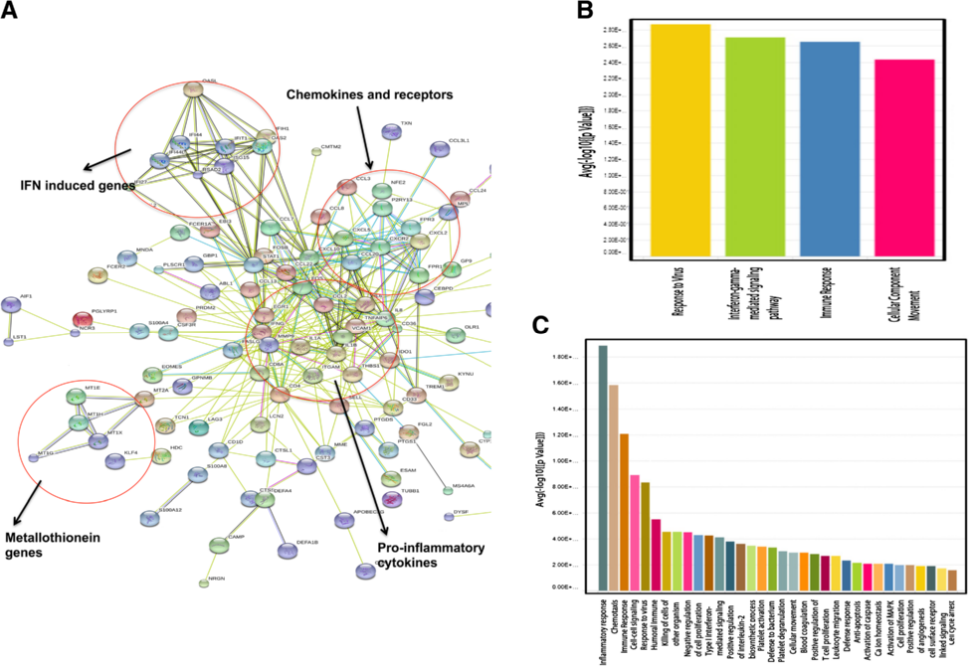
**Predicted interaction networks of genes significantly dysregulated in HVL compared to Control.** (**A**) The interactions between genes were identified using STRING software. Colored lines indicate different sources of evidence for each interaction. Circles highlight the predominant clusters within the network. (**B & C**) Gene Ontology Enrichment analysis for biological processes using significantly differentially regulated genes in LVL and HVL groups in comparison with CT group. Genes that are differentially regulated in LVL group relative to the CT yielded 4 significant terms for biological processes (**B**), while those that are differentially regulated in HVL with respect to CT yielded 34 (**C**). Bar graphs are generated using average –lop of p value for each term on y-axis and term name on x-axis. CT, uninfected control; LVL, low viral load group; HVL, high viral load group.

Next, we analyzed these mRNA transcripts using the STRING and DAVID databases, to identify the biochemical pathways in which they are involved. A comparison of the differentially regulated transcripts in LVL group compared to control group did not show significant changes in cellular pathways, whereas a comparison of the control and HVL groups exhibits distinct pathway regulation (Figure 4A). Though the network shows several interaction groups, four major clusters were apparent; this includes cluster 1 (chemokines and its receptors), cluster 2 (proinflammatory cytokines), cluster 3 (Interferon induced genes) and cluster 4 (metallothionein genes). Among these clusters, the most significantly regulated molecules comprise inflammatory factors, cytokines/chemokine, cell cycle and apoptosis related proteins, cell signaling molecules, factors expressed in response to virus/bacterial infection, innate factors and cell-to-cell interaction molecules. Most importantly, many of the inflammatory factors and cytokines (CXCL5, CCL2, CCL8, CXCL10, CCL7, IL1α, IL-1β, IL6 and IL8) are upregulated in the HVL group, whereas antiviral factors and innate immune molecules (members of the defensin family, AIF) were significantly downregulated (Additional file 3: Table S2).

We next assessed the functionality of the significantly dysregulated genes with a focus on those genes that were statistically over represented. An analysis of biological functions, using Gene Ontology Enrichment, shows only four significant functions, including response to virus, IFNγ mediated signaling, immune response, and cellular component movement, for the comparison between LVL and controls (Figure 4B). This contrast with the 34 significant functions, including inflammatory response, chemotaxis, and immune response, produced from the comparison between HVL and the control group (Figure 4C). These functions relate mostly to inflammatory response, chemotaxis, cell signaling, cell killing and immune regulation. In addition to the gene ontology enrichment, our analyses on host cellular pathways using Ingenuity Pathway Analysis (IPA) indicate several pathways that are distinctly regulated by HIV-1 and high viral load (Additional file 5: Table S3). Differentially regulated RNA transcripts from HVL group target primarily apoptosis and DNA damage pathways (6 out of the top ten pathways) in accordance with the loss of CD4, whereas, LVL did not show similar pathways. Overall, these results suggest that HIV-1 infection resulting in high viral load alters several genes and cellular processes related to HIV-1 induced disease progression.

### Computational analyses to identify miRNA-mRNA interactions

**Figure 5:**
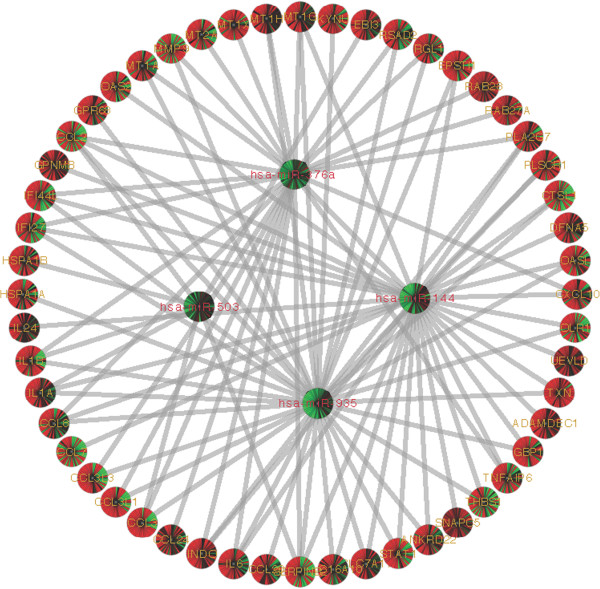
**Network of predicted miRNA-mRNA interactions by GroupMiR using results from miRNA and mRNA array and visualized by Cytoscape.** Regression-based method was used to predict the potential miRNA (circles) that actively regulate mRNA (squares). Selected miRNA (four) were chosen from a list of miRNAs and their targets. Red and green within the mRNA represents up and down regulation, respectively. Each slice within the circle represents HIV-1 infected subject.

MicroRNA binds to the 3’ UTR of the mRNA transcript and regulates gene expression. A single miRNA can potentially regulate single or multiple mRNA and vice versa [57,58]. We used GroupMiR [59], a computational method that integrates miRNA and mRNA expression profiles with miRNA target prediction databases (which rely on sequence analysis), to infer interactions between miRNAs and their target mRNAs. This approach of combining expression and sequence analysis has been shown to reduce the number of false positives in miRNA target prediction. These analyses identified 204 miRNAs and 267 mRNAs, plus 572 possible combination pairs with a score ranging from 0.1004 to 0.9832. The data were used to generate the entire interactome presented in Additional file 6: Figure S3. Further analyses with a few selected pairs indicate that a number of miRNAs (miR-144, miR-376a, miR-503 and miR-935) target a cluster of mRNA including chemokines (CCL2, CCL3, CCL8, IL1β, IL6, PLA2G7, DFNA and members of metallothionein gene family), that are part of the inflammatory response (Figure 5). These results suggest that multiple miRNAs could potentially target the same mRNA transcripts with varying strengths (posterior probabilities). For instance, miR-144 and miR-376a exhibit a score of >0.6 for the inflammatory gene cluster, whereas, miR-503 and miR-935 exhibit a score of <0.2 for the same mRNA transcript, indicating difference in strength of miRNA-mRNA interaction pairs. However, it is important to note that though the strengths are different for these miRNA-mRNA pairs, the inverse correlation between the miRNA and mRNA are constant. The four miRNAs shown in Figure 5 are significantly downregulated (<2-fold, p < 0.05) in HVL samples, whereas 47 of the 50 mRNAs are significantly upregulated (>2-fold, p < 0.05) in HVL subjects. To further verify and correlate the significance of these interactions, we selected a number of mRNA and miRNA pairs and assessed their expression levels. This includes host cellular genes IL6, NRGN (Neurogranin), AIF1, NCR3, ALDH1A1, CCL2 and CCL8 as well as the predicted respective miRNAs. The results presented in Table 3 were derived by comparing the control subjects to HIV-1 positive subjects (LVL and HVL). First we compared the correlative effects of the miRNA and mRNA in LVL and HVL groups compared to control group. The selected mRNA-miRNA pairs showed a significant inverse correlation in their expression pattern, except for CCL8-miR-512-3p and CCL8-miR-518d-3p (p > 0.05) in HVL comparisons. Prediction analyses identified that NRGN could be potentially regulated by 2 miRNA, whereas CCL2 and CCL8 could be potentially regulated by several miRNAs. Conversely, miR-935 has potential binding sites in IL6, CCL2 and CCL8 transcripts in the 3’ UTR region. These results highlight the ability of GroupMiR to accurately predict interactions with high levels of variations found in *in vivo*. However, additional extensive *in vitro* analyses, beyond the scope of this manuscript, are required to confirm these interactions.

**Table 3:** Predicted miRNA-mRNA interacting pairs by GroupMiR in comparison with miRNA profiling studies

**mRNA-miRNA pairs by GroupMiR**			**Control vs LVL (miRNA)**		**Control vs HVL (miRNA)**		**LVL vs HVL (miRNA)**	
**mRNA**	**miRNA**	**P value**	**Fold Change**	**P value**	**Fold change**	**P value**	**Fold change**	**P value**
AIF1 (-2.67)	miR-483-5p	0.8026	5.969	0.00410**	17.088	0.000000205**	2.863	0.0125*
AIF1 (-2.67)	miR-605	0.9418	1.164	0.871	29.059	0.0000000127**	24.961	0.0000000542**
ALDH1A1 (-2.11)	miR-222	0.2438	1.710	0.229	2.558	0.0611	2.856	0.000129**
ALDH1A1 (-2.11)	miR-155	0.7276	5.183	0.0570	16.778	0.000103**	3.237	0.000000808**
NCR3 (-2.3)	miR-34a	0.1528	1.936	0.547	20.678	0.0000153**	10.681	0.000576**
NCR3 (-2.3)	miR-660	0.119	2.276	0.393	6.452	0.00778**	2.835	0.0233*
NRGN (-4.06)	miR-210	0.9606	2.931	0.162	10.536	0.000121**	3.591	0.00276**
NRGN (-4.06)	miR-564	0.9702	2.467	0.229	11.667	0.00000170**	4.730	0.00307**
IL6 (4.01)	miR-144	0.8496	−1.181	0.855	−25.7093	0.00000508**	−21.786	0.000241**
IL6 (4.01)	miR-376a	0.8812	−1.264	0.697	−2.638	0.0185*	−2.088	0.000000797**
IL6 (4.01)	miR-935	0.227	−1.706	0.378	−22.431	0.0000000179**	−13.141	0.0000135**
CCL2 (4.53)	miR-144	0.1506	−1.181	0.855	−25.709	0.00000508**	−21.786	0.000241**
CCL2 (4.53)	miR-196b	0.1026	2.167	0.121	−1.071	0.898	−2.321	0.00479**
CCL2 (4.53)	miR-376a	0.1272	−1.264	0.697	−2.638	0.0185*	−2.088	0.000000797**
CCL2 (4.53)	miR-503	0.1154	1.624	0.403	−3.256	0.0480*	−5.288	0.00788**
CCL2 (4.53)	miR-590-3P	0.1122	3.077	0.099	−2.00	0.252	−6.150	0.00000113**
CCL2 (4.53)	miR-935	0.1732	−1.706	0.378	−22.431	0.0000000179**	−13.141	0.0000135**
CCL8 (5.11)	miR-25	0.5172	−1.095	0.878	2.893	0.00117**	3.169	0.00781**
CCL8 (5.11)	miR-376a	0.119	−1.264	0.697	−2.638	0.0185*	−2.088	0.000000797**
CCL8 (5.11)	miR-512-3p	0.1184	−1.508	0.609	3.942	0.0298*	5.950	0.00829**
CCL8 (5.11)	miR-518d-3p	0.1302	−1.124	0.892	4.867	0.00491**	5.471	0.00367**
CCL8 (5.11)	miR-935	0.4236	−1.706	0.378	−22.431	0.0000000179**	−13.141	0.0000135**

Fold changes and p-values for the miRNA and mRNA in infected PBMC compared to the uninfected controls determined by RealTime StatMiner. * = p < 0.05.

### Role of CD4 count and viral load on host cellular transcriptome

**Figure 6:**
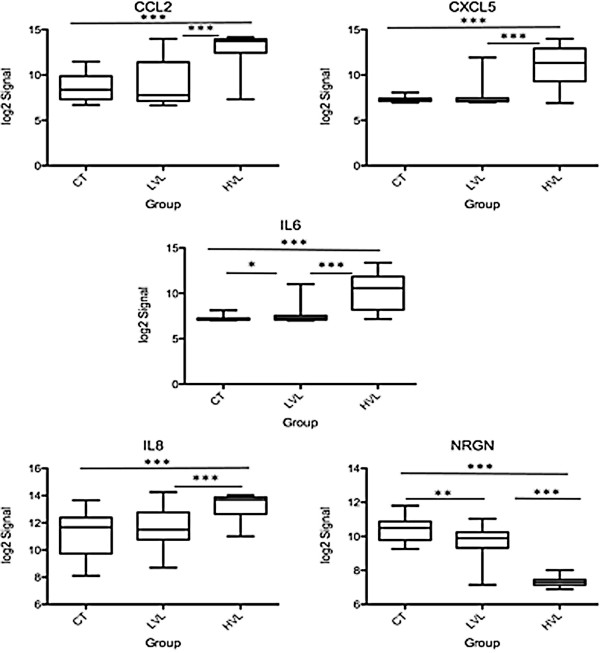
**Expression of selected transcripts among the CT, LVL, and HVL groups.** Unpaired Student’s *t*-test was used to assess significance between the three groups. (* = p < 0.05, ** = p < 0.01, *** = p < 0.0001). CT, uninfected controls; LVL, low viral load group; HVL, high viral load group.

To examine whether other factors, besides the presence of high viral load, are responsible for the differential miRNA and mRNA profiles, we selected a number of cellular factors (CCL2, CXCL5, IL6, IL8 and NRGN) from our predictions and assessed their expression between the three groups. The presence of high viral load significantly influences the expression of CCL2, CCL8, IL6 and IL8, whereas NRGN was inhibited (Figure 6). HIV-1 infection itself down regulated NRGN expression as seen in LVL. Similarly, Defensin and AIF1 were also down regulated by infection and HVL significantly augmented this inhibition (Additional file 3: Table S2). Next, we assessed whether there is a possible correlation with CD4 count, nadir CD4 count, viral load, and host cellular factors (Table 4). As CD4 count is one of the major determinants of HIV infection and plays a role in altering the transcriptome profile [4], we performed Spearman correlations between the CD4 count, nadir CD4 count, viral load and the signal intensity of each selected transcriptome within the three groups of subjects. None of the transcripts significantly correlate with either CD4 count or nadir CD4 count within the uninfected control group (Table 4; panel A). Within the LVL group, IL6 and NRGN significantly correlate with CD4 count (p < 0.01 and p < 0.05) and not with the nadir CD4 count (Table 4; panel B). In the HVL group, IL8 showed a significant correlation with CD4 count (p < 0.05) and nadir CD4 count (p < 0.001), whereas, IL6 correlated with nadir CD4 count only (Table 4; panel C). Next, we assessed, whether the level of viral load within the HVL group is directly or inversely correlated with host cellular factor expression, and results indicate that none of the transcripts significantly correlate with either CD4 count or nadir CD4 count. Together, these results indicate that cellular factors are significantly altered by viral load exceeding 50,000 copies/ml, however the increase in viral copy number (>500,000/ml) did not alter the expression proportionately suggesting a threshold effect.

**Table 4:** A-C: Spearman correlations between selected transcript signals and CD4 count, Nadir CD4 count, and viral load control group

		**CD4 Count**		**Nadir CD4**			
**(A) Control group**	Transcript	Spearman r	p-value	Spearman r	p-value		
	CCL2	−0.00296	0.986	0.156	0.358		
	CXCL5	0.0385	0.821	0.101	0.553		
	IL6	−0.0236	0.877	0.0424	0.799		
	IL8	0.0234	0.891	0.227	0.176		
	NRGN	−0.186	0.278	−0.172	0.309		
		CD4 Count		Nadir CD4			
**(B) LVL group**	Transcript	Spearman r	p-value	Spearman r	p-value		
	CCL2	−0.281	0.133	−0.193	0.283		
	CXCL5	0.025	0.895	−0.026	0.883		
	IL6	−0.488	0.006*	−0.185	0.304		
	IL8	−0.219	0.246	−0.143	0.427		
	NRGN	0.41	0.024*	0.314	0.074		
**(C) HVL group**		CD4 Count		Nadir CD4		Viral load	
	Transcript	Spearman r	p-value	Spearman r	p-value	Spearman r	p-value
	CCL2	−0.022	0.918	−0.084	0.695	0.202	0.355
	CXCL5	−0.277	0.190	−0.367	0.078	0.190	0.385
	IL6	−0.324	0.122	−0.414	0.044*	−0.199	0.364
	IL8	−0.495	0.014*	−0.609	0.001**	−0.085	0.670
	NRGN	0.224	0.292	0.153	0.475	0.143	0.516

Spearman correlations between selected transcripts and CD4 count, Nadir CD4 count, and viral load (where applicable) within the three study groups obtained from GraphPad Prism software set to produce two-tailed p-value. * = p < 0.05; ** = p < 0.001.

### Validation of host cellular factors regulated by HIV-1 at RNA and protein level

**Figure 7:**
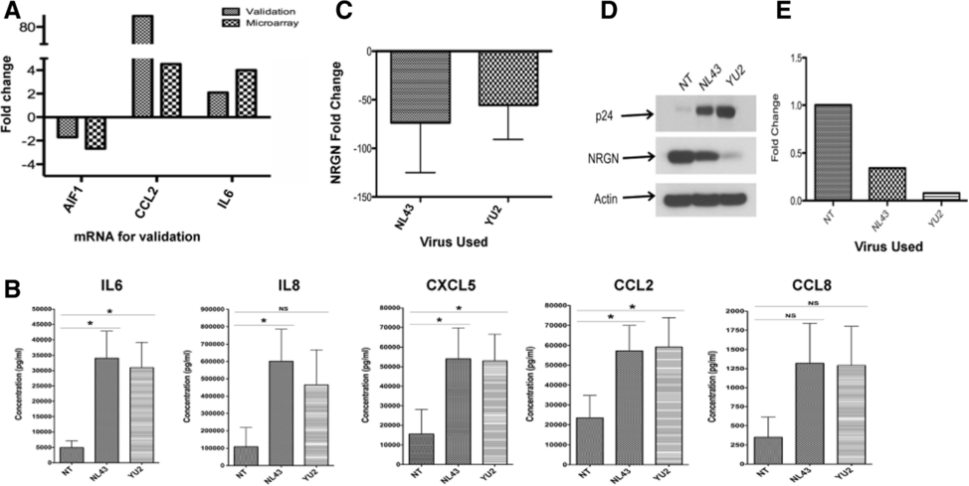
**Validation of HIV-1 regulated host cellular factors at the RNA and protein level.** (**A**) Independent validations of randomly selected differentially regulated mRNAs from transcriptome analyses. qRT-PCR was performed to validate the expression of selected mRNAs using specific primer and probe pairs. Fold increase/decrease was calculated based on normalization to RPLPO. Average fold change for each mRNA represents fold change obtained from independent donors (N = 5 per group). (**B**) Inflammatory factors released by HIV-1 infected PBMC compared to uninfected control cells. Expression of CCL2, CCL8, CXCL5, IL6 and IL8 was monitored by ELISA in supernatants obtained from PBMCs infected with CXCR4-coreceptor utilizing virus (NL43), CCR5-coreceptor utilizing virus (YU2) or mock infected PBMCs (n = 7). * = p < 0.05; NS, Not significant. (**C**) Expression level of NRGN transcript in PBMCs infected with NL43, YU2 or mock infected cells. Post infection, RNA was isolated and qRT-PCR was performed using NRGN specific primers and probe and results were normalized to RPLPO control. Fold change was calculated using uninfected/mock infected PBMC controls. (**D**) Immunoblot of NRGN to measure the protein level in PBMC infected with HIV-1 virus or mock. Gag-p24, represents infectivity; and Actin, represents loading control. (**E**) Densitometry was performed to quantitate the fold change in signal intensity compared to uninfected (NT) and normalized with Actin level. NT, uninfected control; NL4-3, CXCR4 coreceptor virus; and YU2, CCR5 coreceptor virus.

Furthermore, we selected a few mRNAs from the differentially regulated mRNA from Additional file 3: Table S2 (AIF, CCL2 and IL6) and validated them in independent HVL donors (Figure 7A). Results indicate that AIF is downregulated in both the array as well as in qRT-PCR assay, whereas CCL2 and IL6 qRT-PCR showed an upregulation, coinciding with the array data. Although the number of transcripts validated is small, these results suggest that validation of mRNA between the array and qRT-PCR assay are concordant with each other specially in transcriptome profiling.

Although, an *in vivo* validation of serum cytokines and chemokines as well as other markers is ideal, we evaluated the inflammatory factors at the protein level using an *in vitro* experimental culture model. Normal human primary PBMCs were infected with HIV-1 virus and the levels of cytokines/chemokines released post infection were assessed (Figure 7B). Results indicate that HIV-1 infection significantly increased the expression of inflammatory factors and it is independent of viral tropism. Both NL43 and YU2 significantly upregulated the level of IL6 by 8-fold, CCL2 by 3-fold, and CXCL5 by 4-fold with a significance of p < 0.05 in multiple donors. However, the upregulation was about 2.5-fold for CCL8 (p > 0.05) and 8-fold with p < 0.05 for IL8 in NL43 infected PBMCs, whereas it was 3-fold (p > 0.05) in YU2 infected PBMCs. These results exhibit a direct correlation of the mRNA transcript and protein level that showed upregulation in HIV-1 subjects compared to control subjects.

We also tested the expression level of NRGN in these samples by performing qRT PCR. Results indicate that PBMCs infected with NL43 or YU2 showed significant downregulation of the NRGN RNA transcript compared to mock or uninfected control (Figure 7C). To further confirm the expression of NRGN at the protein level, expression of NRGN in cell lysates of PBMCs infected with NL43, YU2 or mock was assessed by immunoblotting using an anti-NRGN antibody (Figure 7D). PBMCs infected with NL43 or YU2 showed significant down regulation of NRGN compared to uninfected or mock-infected control cells. Further quantitative analysis using densitometry scanning indicate that the reduction is >50% in PBMCs infected with NL43 virus, whereas a >80% reduction was found in PBMCs infected with YU2 virus (Figure 7E). It is not clear why the two different viruses exhibit different levels of down regulation of NRGN. One possible explanation could be due to the infectivity level within these cultures, as both macrophages and CD4+T cells are infected by YU2, whereas, NL43 infects only CD4+ T cells.

## Discussion

Obligatory intracellular pathogens, such as viruses, are known for their ability to manipulate host cells both at the molecular and cellular levels. Studies have shown that the host cellular transcriptome is regulated by HIV-1 infection, as part of the cells’ response to pathogen insult [60,61]. HIV-1 infection results in differential regulation of specific cellular genes in target cells such as CD4+ T cells and monocytes/macrophages suggesting that virus infection alters host cellular proteins either to evade the immune system and/or for optimal viral replication. Considering the prominent role played by miRNA in the regulation of gene expression, we hypothesized that analysis of miRNA in samples from HIV-1 infected individuals may provide information about the molecular network and correlate with the disease status in the host. The novel features of our present study are the following: (i) We have utilized samples from HIV-1 infected hosts characterized for viral load (HVL and LVL), together with appropriate age matched controls; (ii) The samples were derived from a large number of HIV-1 infected subjects; (iii) ours is one of the first *in vivo* studies to compare miRNA and mRNA simultaneously in PBMCs isolated from a cohort of HIV-1 infected and uninfected subjects. Our results show that HIV-1 regulates the cellular transcriptome significantly through miRNAs, resulting in an inverse correlation of miRNA-mRNA expression. Furthermore, we have also established that there is an interrelationship between multiple miRNA and mRNA interactions and vice versa within the infected individuals.

The majority of miRNAs are transcribed from noncoding DNA, however a number of miRNAs are also known to be expressed along with mRNA suggesting a specific role for mRNA regulation [62-64]. Our comparative analysis of miRNA and mRNA indicate that HIV-1 infection alters both miRNA and mRNA expression profiles in HIV-1 infected subjects compared to uninfected controls. However, the dysregulation of both the RNA species is less prominent in LVL subjects compared to the HVL subjects indicating that viral load has a significant effect. Differentially expressed miRNA between groups indicates both overlapping and group specific miRNA expression. For instance, HIV-1 infection independent of viral load altered expression of only 4 miRNAs, whereas 191 miRNAs are significantly regulated in the high viral load group compared to uninfected controls. Previous studies using PBMCs or CD4+ T cells from infected viremic, elite controllers and multiply exposed uninfected (MEU) subjects indicate that HIV-1 infection and/or virus exposure resulted in signature miRNA expression profile further supports the role of HIV-1 induced miRNA regulation [41,46,48].

Although a number of commonly dysregulated miRNAs were noted in these studies, there is also a greater degree of variation between these studies including the current study [46,48]. The differences observed between these studies could be due to a number of reasons including array platforms, the patient population, sample size, software used and type of analyses, cut-off set by each group and selection criteria of study subjects. Our study has a large sample size (n = 30 or more) and all of our LVL donors were receiving Highly Active Antiretroviral Therapy (HAART) and had no or undetectable viral load for more than 5 years prior to sample collection. Another possible explanation is the cell types used in these studies (total PBMC vs. purified CD4+ T cells), as these are known to exhibit distinct cell specific miRNA profiles [65,66]. It is also important to point out that our HVL group has very low CD4+ T cell count (<200 cells), suggesting that the differentially regulated miRNAs could be the result of non CD4+ T cell types in response to HIV-1 infection.

Results from our analyses identified several miRNA that are uniquely regulated by HIV-1; however the functional consequences of many of these miRNAs are not fully understood. Further analyses on cell specific miRNAs expressed during virus replication suggest a role for miR-132, -125b, -155, -150, -223 -382 and Let-7 in CD4+ T cells [67-69] and miR-198, -28-3P, 125b, -198, -223 and -382 in monocytes/macrophages [40,70,71], respectively. In our analyses using PBMCs, we noted differential regulation of miR-132, Let-7, -28-3P, -125b, -223, -382, but no difference in others. Additionally, miRNAs (miR-214, -223, 132, -128, -150, -32, 24, -93, -198, -296, 351 and -17-92 cluster) are known to target viral RNA and possess antiviral activity and innate immune functions [72,73]. Among these miRNAs, miR-214 and miR-223 are significantly down regulated in HVL subjects, whereas miR-32, miR-198, miR-128, miR-150 and miR-15 did not show any change. MiR-146a has been linked to innate immunity and immune activation [74,75] and miR-155 is involved in T cell proliferation and Treg regulation [76,77]. These miRNAs are significantly upregulated in HVL subjects compared to seronegative and LVL subjects, suggesting that inflammatory factors are highly upregulated in subjects with HVL supporting hyper activation of immune cells during AIDS. Our studies have shown that the LVL group is similar to the uninfected controls at the mRNA level as evident in few gene interactions in the STRING pathway analysis of the mRNA array data similar to previous studies [42]. On the other hand, several host cellular pathways and networks including cytokine and chemokine interactions, signaling pathways, IFN induced genes, metallothionein genes and pro-inflammatory cytokines were dysregulated in high viral load in comparison to uninfected controls and low viral load samples. Although we have highlighted immune cell related functions in this analyses further analyses indicate that additional pathways such as apoptosis and cell death are that are distinctly regulated in HVL subjects compared to LVL and control further supporting the CD4 loss observed in this group.

Overall, we found only 41 significantly dysregulated mRNAs between uninfected and LVL and all of these mRNAs had a fold change of less than 2. Meanwhile, the comparisons of CT vs. HVL and LVL vs. HVL yielded 309 and 182 significantly regulated probes respectively. Several of these pathways (IFN signaling, innate response, defensins, cytokine/chemokine signaling and T cell signaling) that are dysregulated in the high viral load (compared to low viral load), are consistent with the results of other studies using HIV-1 specific target cell types [4,42-44,78]. We also noticed APOBEC3G, a cellular defense factor against retroviruses, to be upregulated in the presence of infection (CT vs. LVL) and high viral load (CT vs. HVL), which parallels Rotger et al. and their finding that the gene is upregulated with increasing viral load [45]. Although the current study utilized total PBMC for miRNA and mRNA profiling, the results from this analysis reflect both the direct and indirect effect of HIV-1 on miRNA and mRNA expression as well as capturing the dysregulated genes from both monocyte and T cell lineages. As it is well established that less than 1% of the cells in total PBMCs are productively infected by the virus, the significant changes observed here may not be result of a direct effect of HIV-1 infection, but may instead be indirect effects. These indirect effects could be due to nonproductively infected cells, exposure of virus particles (both defective and replication competent) to target and non-target cells as well as cellular and viral factors released from the infected cells that could affect bystander cell population. Our results show that it would certainly be worthwhile to perform cell specific analysis on HIV-1 target cells using a focused array to assess the effect that infection on the RNA species within the individual cell types, but this is beyond the scope of this manuscript.

To study the interaction between the miRNA and mRNA, we chose not to simply rely on online interaction databases, instead, we elected to use our array data as a supplement to GroupMiR. Notably, our analysis has predicted pathway-associated targets for many miRNAs, suggesting that miRNAs effectively target host cellular pathways during infection and disease progression. Although several of the predicted miRNA-mRNA pairs showed inverse correlation, we also observed a few positive correlations in mRNA regulation. Specifically, in case of inflammatory factors, IL6, CCL2 and CCL8 are inversely regulated by miR-144, miR-376a, miR-935 and miR-146a, whereas, in our study both expression of miR-512-3p-CCL8 and miR-518d-3p-CCL8 are positively correlated in HVL subjects compared to uninfected controls. Most importantly, our studies have identified several new miRNAs that could potentially regulate RNA transcripts such as AIF1, ALDH1A1, NCR3 and NRGN that warrant further research. Though it is established that the host transcriptome is regulated by HIV-1, it is not clear how transcription is regulated. Based on our results, it is possible to predict that one of the mechanisms that the virus employs is the use of miRNA, as regulation of a single miRNA could modulate multiple mRNAs that belong to specific pathways, which would be beneficial for the virus.

The presence of high viral load has an impact on several mRNA transcripts, however, the effect of other factors such as CD4 count and nadir CD4 has a significant negative correlation for IL8; this is consistent with data in the literature that associates infection with elevated IL8 [79]. This was not surprising because the nadir CD4 counts for some of the HVL subjects were quite low. Furthermore, this paralleled Stone et al.’s findings of elevated IL6 in HIV patients after an immune restoration [80]. We also found significant correlations within LVL for IL6 and NRGN and CD4. Interestingly, we found no significant correlations with the level of viral load, indicating that the presence of virus has an effect on producing differential mRNA expression but the level of the viral load makes no additional difference. Together these observations suggest that HIV-1 infection, irrespective of its severity, has significant impact on host cellular transcripts and gene expression resulting in immune dysregulation. These findings might provide further explanation on why immune function is never restored back to normal in HAART treated HIV-1 subjects with no detectable virus load. Our study of miRNA and mRNA profiles in individuals infected with low and high levels of viral load has significant clinical relevance. The finding of several miRNAs that are specific to a particular viral load could lead to the use of these miRNAs as biomarkers for the determination of prognosis of the disease.

## Conclusions

In this study, we have shown that the expression profile of miRNA and mRNA in HIV-1 infected individuals with high viral load is very distinct from controls indicating that gene expression is significantly altered in HIV-1 target cells in response to virus replication. We also identified several novel genes whose expression levels vary significantly in our cohorts, and which have potential as therapeutic targets. By using a comparative profiling of miRNA and mRNA simultaneously in these study populations we are able to predict putative miRNA-mRNA interactions and a mathematical model to explain the expression of a given mRNA target in terms of its interaction with the miRNAs thought to regulate its expression. Importantly, we identified several new mRNA targets (Defensin, Neurogranin, AIF) as well as the miRNAs that could be involved in regulating their expression through the miRNA-mRNA interaction. These findings will open new avenues to target host cellular factors using RNAi and anti-miRNA technologies to combat HIV-1.

## Methods

### Study Population and selection criteria

All donor samples were obtained from the Pittsburgh site of the Multicenter AIDS Cohort Study. The study was approved by the Institutional Review Board (IRB) at the University of Pittsburgh and samples were collected using informed consent forms according to the University policies. Donor blood was processed by Ficol-hypaque and PBMCs were stored in RNALater or frozen in LN_2_ for further use. The study population comprised uninfected seronegative controls (N = 36), HIV-1 positive subjects with low viral load (<40 copies/ml) (N = 32), and HIV-1 positive subjects with a high viral load (>50,000 copies/ml) (N = 31) at the time of sample collection. Details of the subjects including age, CD4 counts, nadir CD4 counts, viral load at the time of sample collection, viral load for the past 5 years and number of years in ART/HAART are included in Table 5.

**Table 5:** Clinical status, viral load, CD4 counts of subjects used in this study

**Parameters**	**Control HIV-1 negative**	**HIV-LVL (HIV-1- low viral load)**	**HIV-HVL (HIV-1 high viral load)**
Number per group	36	32	31
Years post SC	N/A	6-26	0-11
Age median (range)	50.5 (28-74)	51 (34-62)	42 (28-65)
CD4 count range (time of sample)	385-2194	193-1421	38-378
Nadir CD4 median (range)	562 (240-1074)	259.5 (13-639)	188 (29-469)
Viral load (time of sample collection)	N/A	<40	46,053-561,627
Viral load variations for past 5 years	N/A	Remain <40	High
Years on HAART/ART	N/A	2-14	0-7

### MicroRNA profiling and data analysis

Total PBMCs were used for RNA isolation. Total RNA was isolated using the MirVANA kit (Applied Biosystems), as suggested by the manufacturer. RNA quality was determined using the Nanodrop2000 spectrophotometer and Bioanalyzer and samples with high RNA quality (RIN values between 8 and 9.7) were used for further profiling. The human microRNA microfluidic card set v3.0 (Applied Biosystems) was used for miRNA profiling of samples. This set enables quantitation of 768 miRNA in total, which include 754 targets, 4 endogenous controls (MammuU6/U6 snRNA run in quadruplicates, and RNU44, RNU48, and ath-miR-159a run in duplicates). One μg of RNA was reverse transcribed using *Taqman microRNA reverse transcription kit (Applied Biosystems, CA) along with megaplex primer pools, human pools set v3.0; the resulting PCR product was loaded on to array cards with Taqman Universal Master Mix II-No UNG, which were run on *ViiA7 Real-Time PCR system according to manufacturer’s protocol as well as standardized protocols developed in our laboratory.

Initial miRNA expression data was analyzed using integrated ViiA7 software (Applied Biosystems). Each run was exported individually using the auto threshold and was then uploaded to RealTime StatMiner software (Integromics, PA) for further analysis. Detectability threshold for miRNA assays was set to Ct value less than or equal to 36 (as no preamplification step was included) in more than 25% of all samples in each group. We used Grubbs’ method to eliminate outliers within technical replicates and imputed missing Ct values based on an aggregation to the median. We used the Genorm method to select the endogenous control U6-snRNA/MammU6 for normalization based on stability scoring across the samples in each group. The aggregation method selected was to the median. Differentially expressed miRNA between different groups were identified using parametric *t*-test or LIMMA (one factor analysis) and were then sorted using the Benjamini-Hochberg false discovery rate (FDR) method with adjusted p-values <0.05 and p < 0.01. The fold changes were obtained using linear RQ values. Hierarchical clustering for differentially expressed miRNA was performed with their corresponding dCt values across the samples in different groups with ‘Complete linkage’ clustering method and ‘Euclidean’ distance measure for dendrograms.

### mRNA profiling and data analysis

For whole genome transcriptome analysis, we used Illumina HT-12 V4 array bead chips (Illumina, Inc., San Diego, CA, USA) for mRNA profiling of the different groups (Control, LVL and HVL) in the study. Each array targets about 47,231 probes that include 28,688 well-characterized or annotated coding transcripts along with 11,121 coding transcripts with provisional annotation and remaining being non-coding transcripts and splice variants. RNA samples (1μg) were labeled using the ‘TotalPrep RNA’ labeling kit (Ambion), reverse transcribed to cDNA, hybridized onto array bead chips overnight on rocker and scanned on ‘iScan system’ according to the manufacturer's protocols as well as standardized protocols developed by the Genomics and Proteomics Core Laboratories at the University of Pittsburgh. Datasets will be deposited in NCBI gene expression and hybridization array data repository GEO database.

### Statistical analysis

Data analysis was performed using the Illumina BeadStudio software to delineate the false discovery rate (FDR) and differences with statistical significance (p < 0.05). Initial raw data analysis and cubic spline normalization was done using the BeadStudio Gene expression module (Illumina, Inc.). The normalized sample probe profile and control probe profile were then uploaded to Integrated Biomarker Discovery (IBD) (Integromics) for further data analysis. A total of 21,852 probe sets were detected in all samples of different groups (Control, LVL and HVL) with present call filter set to probes detected in > 75% of samples in each group. Linear models for microarray data (LIMMA) (one factor analysis) was used to identify differentially expressed genes/probes between different groups. These genes were further sorted based on the Benjamini-Hochberg false discovery rate (FDR) method with an adjusted p-value <0.05 and fold change cut-off of at least 2-fold up/down regulation. Hierarchical clustering for differentially expressed genes/probes was performed using their corresponding detection signal values across the samples in different groups with ‘Complete linkage’ clustering method and ‘Euclidean’ similarity measure for dendrograms. Statistical analyses for *in vitro* experiments and figures were generated using GraphPad Prism.

### Validation of differentially regulated miRNA and mRNA

Based on the data analyses, selected miRNA and mRNA were verified by qRT-PCR. RNA samples from independent subjects (n = 5) were used to validate the high throughput microarray results using miRNA and mRNA specific primers and probes as suggested by the manufacturer (Applied Biosystems). Following normalization to U6 (miRNA) or RPLPO (mRNA), the data were exported and analyzed in RealTime Statminer software, as suggested by the manufacturer.

### Pathway analysis

To determine gene interactions and correlation networks, we used IPA and STRING. The Gene Ontology Enrichment (GO) analysis tool within IBD was used to detect biological annotations that are statistically over-represented in the list of differentially regulated genes/probes between different groups (e.g. Control and LVL). With minimum number of overlapping genes equal to 3 and FDR threshold of <0.05, both singular and concurrent enrichment analysis were carried out. Significant terms for biological processes (BP), molecular functions (MF) and cellular components (CC) were retrieved, arranged in an order based on p-value for corresponding term.

### Interaction of miRNA-mRNA using GroupMiR

We used GroupMiR [59], a computational method that integrates miRNA and mRNA expression profiles with miRNA target prediction databases (which rely on sequence analysis), to infer interactions between miRNAs and their target mRNAs. This approach of combining expression and sequence analysis has been shown to reduce the number of false positives in miRNA target prediction. We downloaded the predictions from MicroCosm database (http://www.ebi.ac.uk/enright-srv/microcosm/htdocs/targets/v5/) and used these as priors for our method. We used the default parameters of GroupMiR for this analysis [59]. The analysis was done with HVL and LVL samples where paired miRNA and mRNA expression profiles are available. The log10 ratios of the mRNA expression levels of HVL/LVL samples to the average values of control samples were used. For miRNAs, the log10 ratios of the average values of control samples to the dCT values of HVL/LVL samples were used.

### Biological validation of HIV-1 regulated factors *in vitro*

HIV-1 induced cytokines and chemokines were tested in an *in vitro* model by infecting normal donor blood derived PBMCs infected with CXCR4 - or CCR5 coreceptor utilizing viruses (NL43 and YU2 respectively) as described [81,82]. Infection was confirmed via a HIV-1 p24 ELISA. Seven days post infection, the supernatant was assessed for the production of CCL2, CCL8, IL6 and IL8 by ELISA (BD Biosciences, CA) and RNA and cell lysates from infected cells was assessed for NRGN by qRT-PCR and western blot, respectively [83].

## Competing interests

The authors declare that they have no competing interests.

## Authors’ contributions

KD, PN and PI performed miRNA and mRNA arrays and analyzed the data; KD, PN, HL, AT and ZB performed the bioinformatics analyses; VA, JM and KD wrote the manuscript; CR critically revised the manuscript; PN and WB coordinated the study; All authors read and approved the final manuscript.

## Pre-publication history

The pre-publication history for this paper can be accessed here:

http://www.biomedcentral.com/1471-2334/13/250/prepub

## Supplementary Material

Additional file 1: Table S1 Differentially regulated miRNA in low and high viral load groups compared to uninfected control group using. Click here for file

Additional file 2: Figure S1 Hierarchical clustering of miRNAs in HIV-1 infected low viral load (LVL) versus high viral load (HVL) subjects. The dendogram depicting the clustering of samples is calculated using Complete linkage with Euclidian distance measure values. Color ranging from green to red indicates minimum to maximum dCT. Numbers on Xaxis represent subject group. The clustergrams were generated using data from StatMiner analyses. Click here for file

Additional file 3: Table S2 Differentially regulated mRNA probes in low (LVL) and high viral load (HVL) groups compared to uninfected control group with statistical significance. Click here for file

Additional file 4: Figure S2 Hierarchical clustering of differentially regulated mRNA in low viral load (LVL) ompared to high viral load (HVL) subjects. The probes in this clustergram are significantly differentially regulated (p < 0.05). Red indicates high, blue indicates low, and gray stands for no change in level of expression. Numbers on X-axis represent subject group; Y-axis represents the gene symbol. Click here for file

Additional file 5: Table S3 **a**: Top ten Canonical pathways representing mRNA that are differentially expressed in LVL group compared to uninfected seronegative subjects. **b:** Top ten Canonical pathways representing mRNA that are differentially expressed in HVL group compared to uninfected seronegative subjects. Click here for file

Additional file 6: Figure S3 Network of predicted miRNA-mRNA interactions by GroupMiR using results from miRNA and mRNA array and visualized by Cytoscape. Regression-based method was used to predict the potential miRNA (circles) that actively regulate mRNA (squares). Differentially regulated miRNA and mRNA in HIV-1 infected subjects compared to the uninfected controls were used to predict the miRNA-mRNA pairs. Red and green within the mRNA represents up and down regulation, respectively. Each slice within the circle represents HIV-1 infected subject. Click here for file
