# Supplementary material for: MicroRNA regulation and its effects on cellular transcriptome in Human Immunodeficiency Virus-1 (HIV-1) infected individuals with distinct viral load and CD4 cell counts
Source: BMC Infect Dis. 2013 May 30;13:250. doi: 10.1186/1471-2334-13-250 (PMC3680326; doi:10.1186/1471-2334-13-250)
Supplement: Additional file 1: Table S1 — Differentially regulated miRNA in low and high viral load groups compared to uninfected control group using. [file 1471-2334-13-250-S1.pdf]

**Table S1: Differentially regulated miRNA in low and high viral load groups compared to uninfected control group u**

## Control vs. Low viral load

| miRNA | p-value | Fold Change |
|---|---|---|
| hsa-let-7f-2#-002418 | 1.46E-02 | 6.7013 |
| hsa-let-7i#-002172 | 4.11E-02 | 3.6956 |
| hsa-miR-1183-002841 | 2.80E-02 | 4.5678 |
| hsa-miR-124#-002197 | 4.35E-04 | 248.4524 |
| hsa-miR-1250-002887 | 2.80E-02 | -10.6284 |
| hsa-miR-1257-002910 | 4.00E-02 | 7.7877 |
| hsa-miR-1260-002896 | 3.99E-02 | 5.6598 |
| hsa-miR-1262-002852 | 4.13E-04 | -1117.8098 |
| hsa-miR-1275-002840 | 2.84E-02 | 6.2628 |
| hsa-miR-1291-002838 | 1.46E-02 | 5.1285 |
| hsa-miR-1303-002792 | 2.47E-02 | 7.4270 |
| hsa-miR-1304-002874 | 4.61E-06 | 113010.9935 |
| hsa-miR-138-2#-002144 | 2.27E-03 | 8.7629 |
| hsa-miR-146a#-002163 | 2.58E-02 | -23.2904 |
| hsa-miR-147-4373131 | 9.45E-03 | -7.2747 |
| hsa-miR-148b#-002160 | 1.30E-02 | 3.5335 |
| hsa-miR-151-3p-002254 | 3.99E-02 | 3.5268 |
| hsa-miR-16-4373121 | 4.13E-02 | 14.2221 |
| hsa-miR-18b#-002310 | 2.58E-02 | -27.6037 |
| hsa-miR-191#-002678 | 1.63E-02 | 4.4852 |
| hsa-miR-194#-002379 | 2.27E-03 | 13.6655 |
| hsa-miR-21#-002438 | 2.27E-03 | 6.6638 |
| hsa-miR-219-1-3p-4395 | 4.01E-02 | 3.6289 |
| hsa-miR-220b-4395317 | 2.24E-02 | 37.1120 |
| hsa-miR-376b-4373196 | 1.46E-02 | 10.7415 |
| hsa-miR-454#-001996 | 1.82E-03 | 3.1752 |
| hsa-miR-483-5p-439544 | 4.10E-03 | 5.9692 |
| hsa-miR-524-5p-439517 | 0.00887708 | 10.0835 |
| hsa-miR-542-5p-439535 | 2.47E-02 | 4.6363 |
| hsa-miR-548c-3p-43809 | 1.46E-02 | -5.9144 |
| hsa-miR-550-002410 | 4.10E-03 | 4.0567 |
| hsa-miR-584-001624 | 6.72E-03 | 13.7803 |
| hsa-miR-649-001602 | 1.40E-02 | 8.1867 |
| hsa-miR-650-001603 | 3.15E-02 | 5.9026 |
| hsa-miR-744#-002325 | 1.46E-03 | 5.0088 |
| hsa-miR-92b#-002343 | 1.17E-02 | 5.6695 |
| hsa-miR-93#-002139 | 2.84E-02 | 3.0965 |
| hsa-miR-938-002181 | 2.60E-04 | 20.48072181 |
| hsa-miR-941-002183 | 4.59E-02 | 3.969513229 |
| rno-miR-29c#-001818 | 4.00E-02 | 3.972610169 |
| rno-miR-7#-001338 | 3.02E-02 | 5.602637467 |

## Control vs. How viral load

| miRNA | p-value | Fold Change |
|---|---|---|
| dme-miR-7-000268 | 1.26E-07 | 13.87493014 |
| has-miR-1305-002867 | 5.30E-11 | 4078.263417 |
| has-miR-155-4395459 | 1.03E-04 | 16.77843025 |
| hsa-let-7d-4395394 | 2.03E-02 | 4.085508578 |
| hsa-let-7g-4395393 | 1.19E-02 | 8.761654982 |
| hsa-miR-1-4395333 | 4.02E-02 | 6.882940744 |
| hsa-miR-106b#-002380 | 3.86E-02 | 2.748504713 |
| hsa-miR-10a-4373153 | 1.58E-03 | 4.581427036 |
| hsa-miR-10b#-002315 | 4.80E-02 | 2.53059669 |
| hsa-miR-1180-002847 | 0.01589604 | 3.43495718 |
| hsa-miR-1183-002841 | 2.17E-06 | 12.66847663 |
| hsa-miR-1201-002781 | 1.12E-03 | 5.399767917 |
| hsa-miR-1225-3P-002766 | 4.67E-13 | 26.45485442 |
| hsa-miR-1227-002769 | 1.49E-02 | 3.83979813 |
| hsa-miR-124-4373295 | 1.58E-10 | 63.94441338 |
| hsa-miR-1245-002823 | 1.42E-03 | 16845.46439 |
| hsa-miR-1247-002893 | 5.57E-04 | 49.53915351 |
| hsa-miR-1253-002894 | 1.45E-07 | 17.03468336 |
| hsa-miR-1254-002818 | 1.21E-04 | 4.637792173 |
| hsa-miR-1255B-002801 | 8.44E-04 | 4.921461872 |
| hsa-miR-125b-1#-002378 | 0.022202 | 4.461441295 |
| hsa-miR-1260-002896 | 9.72E-03 | 5.8778463 |
| hsa-miR-1262-002852 | 2.66E-02 | -6.03E+01 |
| hsa-miR-1267-002885 | 2.62E-02 | -2.01E+01 |
| hsa-miR-127-3p-4373147 | 2.89E-02 | 3.184511612 |
| hsa-miR-1270-002807 | 3.39E-03 | 5.801833768 |
| hsa-miR-1271-002779 | 1.18E-04 | 3.039572182 |
| hsa-miR-1274A-002883 | 6.05E-05 | 4.466481817 |
| hsa-miR-1275-002840 | 7.47E-05 | 17.07456456 |
| hsa-miR-1276-002843 | 1.96E-06 | 10.48095323 |
| hsa-miR-1282-002803 | 6.59E-04 | 4.775920011 |
| hsa-miR-1283-002890 | 1.03E-02 | 5.667040929 |
| hsa-miR-1284-002903 | 3.69E-05 | 50132.86392 |
| hsa-miR-1285-002822 | 5.15E-03 | 10.09637943 |
| hsa-miR-1290-002863 | 4.19E-05 | 5.121944982 |
| hsa-miR-1291-002838 | 4.33E-04 | 4.347958775 |
| hsa-miR-1298-002861 | 1.78E-03 | 18.83721058 |
| hsa-miR-1300-002902 | 1.50E-03 | 4.714974603 |
| hsa-miR-1303-002792 | 4.67E-13 | 137.2051535 |
| hsa-miR-130b-4373144 | 1.48E-02 | 2.550489557 |
| hsa-miR-130b#-002114 | 1.05E-04 | -7.371873741 |
| hsa-miR-132-4373143 | 3.14E-07 | 12.91743135 |
| hsa-miR-134-4373299 | 3.38E-02 | -2.737199743 |
| hsa-miR-135b-4395372 | 5.43E-03 | 4.194801661 |
| hsa-miR-138-2#-002144 | 7.47E-05 | 14.75599593 |
| hsa-miR-140-5p-4373374 | 1.19E-02 | 3.043312478 |
| hsa-miR-142-3p-4373136 | 6.03E-03 | 10.84376544 |
| hsa-miR-143#-002146 | 9.18E-03 | -5.76E+01 |

| | | |
|---|---|---|
| hsa-miR-144-002676 | 5.08E-06 | -2.57E+01 |
| hsa-miR-146a-4373132 | 9.83E-03 | 10.80949113 |
| hsa-miR-146a#-002163 | 0.04632034 | -6.126977814 |
| hsa-miR-146b-3p-439547 | 2.31E-05 | 6.026408157 |
| hsa-miR-148a#-002134 | 2.41E-02 | -8.98E+01 |
| hsa-miR-148b#-002160 | 7.62E-03 | 3.112987387 |
| hsa-miR-151-5P-002642 | 1.50E-03 | 6.110985331 |
| hsa-miR-152-4395170 | 1.90E-03 | 5.262731632 |
| hsa-miR-155#-002287 | 1.78E-05 | 7.634857628 |
| hsa-miR-15a-4373123 | 2.23E-02 | 4.920624402 |
| hsa-miR-16-4373121 | 1.20E-02 | 16.03235967 |
| hsa-miR-181a-4373117 | 2.26E-03 | 7.415476638 |
| hsa-miR-181c-4373115 | 0.01298629 | 2.897475766 |
| hsa-miR-1826-002873 | 1.08E-02 | -3.98E+01 |
| hsa-miR-185-4395382 | 0.01014398 | 4.102689273 |
| hsa-miR-186-4395396 | 1.24E-02 | 3.861430231 |
| hsa-miR-18a#-002423 | 2.02E-02 | 2.405851326 |
| hsa-miR-18b#-002310 | 2.14E-02 | -1.58E+01 |
| hsa-miR-191#-002678 | 3.49E-03 | 5.955901492 |
| hsa-miR-193a-5p-439539 | 2.17E-06 | 13.71013761 |
| hsa-miR-193b-4395478 | 1.50E-03 | 6.808706875 |
| hsa-miR-194-4373106 | 2.31E-03 | 4.86958654 |
| hsa-miR-195-4373105 | 2.46E-02 | 3.99362515 |
| hsa-miR-19a-4373099 | 2.37E-02 | 7.675342781 |
| hsa-miR-19b-1#-002425 | 1.00E-02 | 2.760137509 |
| hsa-miR-19b-4373098 | 1.28E-02 | 13.55178785 |
| hsa-miR-200b-4395362 | 4.10E-03 | 4.735897947 |
| hsa-miR-200c-4395411 | 3.66E-02 | 3.789084891 |
| hsa-miR-202-4395474 | 9.92E-04 | 5.237766018 |
| hsa-miR-206-000510 | 1.16E-03 | 3.751404947 |
| hsa-miR-20a-4373286 | 1.08E-02 | 10.18643945 |
| hsa-miR-20b-4373263 | 1.97E-02 | 3.318840304 |
| hsa-miR-21-4373090 | 3.51E-06 | 6.153184848 |
| hsa-miR-21#-002438 | 4.03E-05 | 7.890314935 |
| hsa-miR-210-4373089 | 1.21E-04 | 10.53561414 |
| hsa-miR-211-4373088 | 1.67E-02 | 3.032928462 |
| hsa-miR-212-4373087 | 7.74E-08 | 7.200668369 |
| hsa-miR-214-4395417 | 2.77E-02 | -5.073381256 |
| hsa-miR-219-5p-4373080 | 3.22E-02 | 5.070692526 |
| hsa-miR-22-4373079 | 4.47E-05 | 15.41125223 |
| hsa-miR-22#-002301 | 4.08E-02 | 1.767145887 |
| hsa-miR-220c-4395322 | 8.59E-06 | 35101.94716 |
| hsa-miR-221#-002096 | 1.81E-02 | 4.24639961 |
| hsa-miR-222-4395387 | 1.70E-06 | 4.886975108 |
| hsa-miR-223-4395406 | 5.58E-03 | -2.053736221 |
| hsa-miR-24-2#-002441 | 1.70E-02 | 2.785808093 |
| hsa-miR-25-4373071 | 1.17E-03 | 2.892958643 |
| hsa-miR-27a-4373287 | 4.32E-02 | 2.42853155 |
| hsa-miR-27a#-002445 | 1.13E-03 | 2.029079327 |
| hsa-miR-28-3p-4395557 | 7.98E-04 | 1.956747885 |
| hsa-miR-28-5p-4373067 | 8.00E-03 | 3.894108308 |
| hsa-miR-29a-4395223 | 3.98E-02 | 2.907401442 |
| hsa-miR-29a#-002447 | 2.66E-02 | 2.855672718 |

| | | |
|---|---|---|
| hsa-miR-29b-1#-002165 | 1.19E-04 | 7.589387316 |
| hsa-miR-29b-2#-002166 | 6.75E-03 | 6.468322348 |
| hsa-miR-29b-4373288 | 2.07E-02 | 3.555525305 |
| hsa-miR-302a#-002381 | 5.15E-03 | 5.623309117 |
| hsa-miR-302d#-002120 | 3.73E-04 | 24.89965354 |
| hsa-miR-30a-5p-000417 | 4.08E-02 | 3.790306502 |
| hsa-miR-30b-4373290 | 1.83E-02 | 7.280247288 |
| hsa-miR-30c-2#-002110 | 2.17E-06 | 15966.80801 |
| hsa-miR-31#-002113 | 1.97E-02 | -2.718469803 |
| hsa-miR-320B-002844 | 4.38E-03 | 6.78329652 |
| hsa-miR-324-3p-4395272 | 5.61E-04 | 5.661842061 |
| hsa-miR-324-5p-4373052 | 2.28E-03 | 5.166228874 |
| hsa-miR-330-3p-4373047 | 4.43E-03 | 3.603381132 |
| hsa-miR-331-3p-4373046 | 3.19E-02 | 4.597970825 |
| hsa-miR-335#-002185 | 3.04E-03 | 4.615719507 |
| hsa-miR-337-5p-4395267 | 2.37E-02 | 3.097516585 |
| hsa-miR-338-5P-002658 | 2.41E-04 | 136.4889816 |
| hsa-miR-339-5p-4395368 | 3.72E-03 | 3.323813895 |
| hsa-miR-33a#-002136 | 1.81E-02 | 2.468964194 |
| hsa-miR-342-3p-4395371 | 4.08E-02 | 6.912106508 |
| hsa-miR-342-5p-4395258 | 9.42E-05 | 9.713559075 |
| hsa-miR-346-4373038 | 1.70E-06 | 22.98164868 |
| **hsa-miR-34a-4395168** | 1.53E-05 | 20.67824891 |
| hsa-miR-34a#-002316 | 4.02E-02 | 3.039632595 |
| hsa-miR-34b-002102 | 4.29E-04 | 3.23882279 |
| hsa-miR-361-3p-002116 | 1.30E-02 | 3.749723066 |
| hsa-miR-361-5p-4373035 | 2.62E-02 | 2.807058518 |
| hsa-miR-362-5p-4378092 | 4.09E-02 | 2.870256481 |
| hsa-miR-363-4378090 | 4.91E-03 | 3.354433159 |
| hsa-miR-375-4373027 | 2.37E-02 | -3.152605833 |
| hsa-miR-376a-4373026 | 1.85E-02 | -2.638532334 |
| hsa-miR-376b-4373196 | 4.51E-02 | 2.941008912 |
| hsa-miR-378-000567 | 1.21E-04 | 6.368024463 |
| hsa-miR-380-5p-000570 | 1.90E-03 | 7.670777208 |
| hsa-miR-381-4373020 | 9.03E-05 | 7.343627248 |
| hsa-miR-382-4373019 | 5.82E-04 | 7.736485404 |
| hsa-miR-424-4373201 | 1.45E-03 | 4.817213696 |
| hsa-miR-425-4380926 | 7.25E-03 | 6.524155727 |
| hsa-miR-433-4373205 | 1.70E-06 | 14.27815737 |
| hsa-miR-451-4373360 | 1.19E-02 | -4.362941336 |
| hsa-miR-483-5p-4395449 | 2.05E-07 | 17.08813375 |
| hsa-miR-486-3p-4395204 | 4.08E-02 | 3.361467958 |
| hsa-miR-487a-4378097 | 4.02E-02 | 2.507611098 |
| hsa-miR-488-4395468 | 0.01614418 | 3.553588546 |
| hsa-miR-490-3p-4373215 | 5.64E-06 | 18.23678574 |
| hsa-miR-491-5p-4381053 | 2.77E-03 | 3.053787051 |
| hsa-miR-500-4395539 | 1.76E-04 | 3.926214216 |
| hsa-miR-501-5p-4373226 | 3.32E-03 | 5.65556144 |
| hsa-miR-502-5p-4373227 | 1.85E-03 | 3.471441708 |
| hsa-miR-503-4373228 | 4.80E-02 | -3.256553387 |
| hsa-miR-505-4395200 | 3.36E-02 | 3.307064251 |
| hsa-miR-509-5p-4395346 | 5.87E-03 | 5.46227271 |
| hsa-miR-511-4373236 | 1.38E-04 | 8.235456181 |

| | | |
|---|---|---|
| hsa-miR-512-3p-4381034 | 2.98E-02 | 3.942236524 |
| hsa-miR-513C-002756 | 4.43E-03 | 6.083801086 |
| hsa-miR-516-3p-001149 | 3.38E-02 | 2.948377281 |
| hsa-miR-516b-4395172 | 1.75E-06 | 10.1034214 |
| hsa-miR-518b-4373246 | 1.54E-11 | 32.72885076 |
| hsa-miR-518d-3p-437324 | 4.91E-03 | 4.867234049 |
| hsa-miR-518e-4395506 | 8.69E-03 | 4.284690095 |
| hsa-miR-519e#-001166 | 0.00438143 | 32.1623535 |
| hsa-miR-524-5p-4395174 | 6.37E-03 | 7.667536858 |
| hsa-miR-525-3p-4395496 | 1.10E-02 | 4.05560743 |
| hsa-miR-541-4395312 | 8.65E-03 | 6.521780014 |
| hsa-miR-551b#-002346 | 2.25E-02 | 2.373094766 |
| hsa-miR-552-001520 | 2.77E-02 | -3.749805561 |
| hsa-miR-554-001522 | 1.00E-02 | -3.942699002 |
| hsa-miR-564-001531 | 1.70E-06 | 11.66701669 |
| hsa-miR-571-001613 | 1.45E-07 | 23.50474181 |
| hsa-miR-572-001614 | 2.28E-03 | 5.38342451 |
| hsa-miR-575-001617 | 6.71E-03 | 6.335867279 |
| hsa-miR-579-4395509 | 1.81E-02 | 2.867363702 |
| hsa-miR-584-001624 | 5.32E-05 | 34.84067614 |
| hsa-miR-589-001543 | 9.02E-03 | 3.40504916 |
| hsa-miR-596-001550 | 3.95E-05 | 4.738045703 |
| hsa-miR-598-4395179 | 1.19E-02 | 4.064260377 |
| hsa-miR-605-001568 | 1.27E-08 | 29.05944982 |
| hsa-miR-607-001570 | 5.13E-03 | 4.612443395 |
| hsa-miR-615-3p-4386777 | 1.55E-04 | 51.37002386 |
| hsa-miR-615-5p-4395464 | 0.0278837 | 4.806302391 |
| hsa-miR-621-001598 | 8.01E-03 | 19.25685773 |
| hsa-miR-622-001553 | 1.82E-02 | 6.320200953 |
| hsa-miR-623-001555 | 3.78E-08 | 35.23526181 |
| hsa-miR-626-001559 | 4.97E-02 | -5.613064084 |
| hsa-miR-631-001564 | 6.25E-03 | -9.001941012 |
| hsa-miR-636-4395199 | 0.03251524 | 3.047789873 |
| hsa-miR-638-001582 | 1.84E-05 | 14.71255401 |
| hsa-miR-639-001583 | 1.53E-03 | 5.451818205 |
| hsa-miR-642-4380995 | 7.47E-05 | 3.648423737 |
| hsa-miR-643-001594 | 1.95E-03 | 6.814748402 |
| hsa-miR-644-001596 | 0.01614418 | 4.019401608 |
| hsa-miR-645-001597 | 7.78E-03 | -1.53E+01 |
| hsa-miR-650-001603 | 1.47E-05 | 18.03793605 |
| hsa-miR-652-4395463 | 5.30E-04 | 6.066535113 |
| hsa-miR-654-3p-4395350 | 3.17E-02 | 3.459684983 |
| hsa-miR-655-4381015 | 1.58E-02 | 6.574134967 |
| hsa-miR-658-001513 | 1.00E-03 | 256074.4728 |
| hsa-miR-659-001514 | 1.97E-04 | 25.33061999 |
| hsa-miR-660-4380925 | 7.78E-03 | 6.452092068 |
| hsa-miR-661-001606 | 2.09E-05 | 12.97419819 |
| hsa-miR-668-001992 | 4.32E-03 | -4.471952976 |
| hsa-miR-671-3p-4395433 | 4.08E-02 | 2.612865055 |
| hsa-miR-672-4395438 | 4.47E-07 | 21.23813422 |
| hsa-miR-744#-002325 | 1.58E-03 | 4.235715628 |
| hsa-miR-766-001986 | 2.14E-02 | 2.018251179 |
| hsa-miR-769-5p-001998 | 1.08E-02 | 3.038207553 |

| | | |
|---|---|---|
| hsa-miR-770-5p-002002 | 1.27E-03 | 7.690015783 |
| hsa-miR-886-3p-4395305 | 0.00767989 | 4.26141862 |
| hsa-miR-9-4373285 | 9.42E-05 | 10.66248145 |
| hsa-miR-9#-002231 | 0.04493089 | 1.992987685 |
| hsa-miR-92a-4395169 | 4.29E-03 | 6.816814242 |
| hsa-miR-93-4373302 | 1.00E-02 | 4.213684912 |
| hsa-miR-93#-002139 | 5.51E-03 | 3.270610677 |
| hsa-miR-933-002176 | 1.03E-05 | -3.18E+01 |
| hsa-miR-935-002178 | 1.79E-08 | -2.24E+01 |
| hsa-miR-938-002181 | 2.19E-04 | 10.36194252 |
| hsa-miR-939-002182 | 2.57E-05 | 37.01043571 |
| hsa-miR-99b#-002196 | 3.46E-04 | 5.686864826 |
| rno-miR-29c#-001818 | 1.52E-02 | 3.085864535 |
| rno-miR-7#-001338 | 0.00980813 | 5.9249569 |

ısing two Statminer software with statistical significance.

## Low viral load vs. High viral load

| miRNA | p-value | Fold Change |
|---|---|---|
| dme-miR-7-000268 | 3.62E-03 | 4.1039 |
| has-miR-1305-002867 | 7.97E-12 | 6460.409753 |
| has-miR-155-4395459 | 8.08E-07 | 3.236641822 |
| hsa-miR-103-4373158 | 1.85E-02 | 2.526795861 |
| hsa-miR-106b-4373155 | 2.01E-02 | 2.431595935 |
| hsa-miR-107-4373154 | 1.95E-02 | 3.359706855 |
| hsa-miR-10a-4373153 | 1.59E-04 | 5.45571071 |
| hsa-miR-1183-002841 | 4.45E-02 | 2.773408027 |
| hsa-miR-1201-002781 | 0.008733965 | 2.962047272 |
| hsa-miR-1206-002878 | 6.17E-03 | 30.38398055 |
| hsa-miR-1225-3P-002766 | 3.09E-09 | 18.29619753 |
| hsa-miR-1227-002769 | 2.01E-05 | 4.857376948 |
| hsa-miR-1233-002768 | 1.22E-02 | 3.214222937 |
| hsa-miR-124-4373295 | 5.07E-03 | 10.58794609 |
| hsa-miR-124#-002197 | 7.04E-05 | -3.64E+02 |
| hsa-miR-1245-002823 | 4.31E-04 | 8396.381156 |
| hsa-miR-1247-002893 | 3.31E-04 | 37.27352435 |
| hsa-miR-1248-002870 | 4.27E-02 | 3.028911809 |
| hsa-miR-1253-002894 | 8.31E-03 | 4.798619994 |
| hsa-miR-1254-002818 | 1.39E-02 | 3.189952687 |
| hsa-miR-1262-002852 | 3.40E-02 | 18.53709627 |
| hsa-miR-1267-002885 | 1.25E-02 | -6.91E+01 |
| hsa-miR-127-3p-4373147 | 1.93E-02 | 3.692981118 |
| hsa-miR-1270-002807 | 3.25E-04 | 8.968718696 |
| hsa-miR-1271-002779 | 1.65E-02 | 2.137997236 |
| hsa-miR-1274B-002884 | 9.93E-03 | 2.515736434 |
| hsa-miR-1275-002840 | 0.018152174 | 2.726344656 |
| hsa-miR-1276-002843 | 0.027036811 | 3.231765935 |
| hsa-miR-1282-002803 | 3.53E-05 | 11.32979676 |
| hsa-miR-1284-002903 | 4.83E-03 | 36806.94177 |
| hsa-miR-1303-002792 | 1.18E-04 | 18.47390493 |
| hsa-miR-1304-002874 | 8.96E-05 | -2.55E+04 |
| hsa-miR-130a-4373145 | 1.52E-02 | 3.455599164 |
| hsa-miR-130b-4373144 | 1.52E-02 | 2.321009777 |
| hsa-miR-130b#-002114 | 6.47E-04 | -9.048925905 |
| hsa-miR-132-4373143 | 8.80E-09 | 7.857541172 |
| hsa-miR-1324-002815 | 1.18E-02 | -4.997183674 |
| hsa-miR-134-4373299 | 1.09E-03 | -7.84704976 |
| hsa-miR-135b-4395372 | 4.00E-03 | 3.273375588 |
| hsa-miR-140-5p-4373374 | 3.38E-02 | 2.240430968 |
| hsa-miR-142-3p-4373136 | 2.20E-03 | 4.096011999 |
| hsa-miR-143#-002146 | 4.58E-03 | -1.06E+01 |
| hsa-miR-144-002676 | 2.41E-04 | -21.7712014 |
| hsa-miR-144#-002148 | 1.22E-02 | -3.957074571 |
| hsa-miR-146a-4373132 | 2.28E-02 | 1.841304579 |
| hsa-miR-146a#-002163 | 3.46E-02 | 3.801279833 |
| hsa-miR-146b-3p-439547 | 9.63E-04 | 5.38274824 |
| hsa-miR-147-4373131 | 4.73E-02 | 3.058556428 |

| | | |
|---|---|---|
| hsa-miR-148b-4373129 | 1.64E-02 | 2.344156497 |
| hsa-miR-151-5P-002642 | 4.22E-02 | 1.92641438 |
| hsa-miR-152-4395170 | 5.44E-04 | 4.233219469 |
| hsa-miR-15a#-002419 | 4.78E-03 | -3.232795969 |
| hsa-miR-181a-4373117 | 1.53E-03 | 3.159795141 |
| hsa-miR-181c-4373115 | 4.27E-02 | 2.291146637 |
| hsa-miR-1825-002907 | 1.22E-02 | 18.09063636 |
| hsa-miR-1826-002873 | 3.40E-02 | -2.756201259 |
| hsa-miR-183#-002270 | 0.049399725 | -4.521519815 |
| hsa-miR-191-4395410 | 5.81E-05 | -2.353713359 |
| hsa-miR-193a-5p-439539[illegible] | 2.56E-04 | 9.871053529 |
| hsa-miR-193b-4395478 | 1.22E-02 | 1.907932654 |
| hsa-miR-193b#-002366 | 2.82E-03 | -7.26499913 |
| hsa-miR-194-4373106 | 3.96E-02 | 2.506272813 |
| hsa-miR-194#-002379 | 0.018152174 | -6.15708637 |
| hsa-miR-196b-4395326 | 4.78E-03 | -2.321038875 |
| hsa-miR-202-4395474 | 2.89E-02 | 3.965904896 |
| hsa-miR-202#-002362 | 3.40E-02 | -2.571330939 |
| hsa-miR-21-4373090 | 2.99E-05 | 5.82454685 |
| hsa-miR-210-4373089 | 2.76E-03 | 3.595133816 |
| hsa-miR-211-4373088 | 4.27E-02 | 2.920936927 |
| hsa-miR-212-4373087 | 1.19E-05 | 5.360547672 |
| hsa-miR-214-4395417 | 2.07E-02 | -7.005410447 |
| hsa-miR-216b-4395437 | 1.42E-02 | -2.07E+01 |
| hsa-miR-219-5p-4373080 | 4.58E-03 | 11.45617899 |
| hsa-miR-22-4373079 | 2.12E-04 | 10.53320028 |
| hsa-miR-220b-4395317 | 2.65E-02 | -2.82E+01 |
| hsa-miR-220c-4395322 | 1.35E-05 | 72645.73094 |
| hsa-miR-221-4373077 | 1.56E-02 | 3.944121406 |
| hsa-miR-222-4395387 | 1.29E-04 | 2.857903385 |
| hsa-miR-223-4395406 | 2.66E-02 | -2.153693457 |
| hsa-miR-224-4395210 | 3.71E-02 | 3.266457916 |
| hsa-miR-24-1#-002440 | 4.27E-02 | 4.830681266 |
| hsa-miR-24-2#-002441 | 1.95E-02 | 2.79290792 |
| hsa-miR-24-4373072 | 4.48E-02 | 1.727617696 |
| hsa-miR-25-4373071 | 7.81E-03 | 3.168867722 |
| hsa-miR-27a-4373287 | 1.19E-02 | 2.903229913 |
| hsa-miR-27a#-002445 | 4.43E-03 | 2.086912741 |
| hsa-miR-28-3p-4395557 | 9.74E-03 | 1.959935207 |
| hsa-miR-28-5p-4373067 | 8.08E-03 | 2.935697214 |
| hsa-miR-298-4395301 | 2.81E-02 | 9.81599444 |
| hsa-miR-29b-1#-002165 | 1.38E-03 | 5.832969458 |
| hsa-miR-29b-2#-002166 | 0.008733965 | 8.439714698 |
| hsa-miR-29b-4373288 | 0.018152174 | 3.670935111 |
| hsa-miR-302a#-002381 | 3.69E-02 | 3.468442529 |
| hsa-miR-302c#-000534 | 1.32E-04 | 46.72734746 |
| hsa-miR-302d#-002120 | 1.42E-02 | 15.01503671 |
| hsa-miR-30b-4373290 | 1.74E-02 | 2.552245456 |
| hsa-miR-30c-2#-002110 | 1.19E-05 | 9831.29403 |
| hsa-miR-30c-4373060 | 1.82E-02 | 2.376015665 |
| **hsa-miR-31#-002113** | 3.55E-03 | -3.599965344 |
| hsa-miR-320B-002844 | 3.27E-02 | 3.658125735 |
| hsa-miR-324-3p-4395272 | 1.34E-03 | 3.799683316 |

| | | |
|---|---|---|
| hsa-miR-324-5p-4373052 | 3.27E-03 | 4.809948595 |
| hsa-miR-328-4373049 | 1.10E-02 | 3.459600668 |
| hsa-miR-330-3p-4373047 | 7.46E-04 | 3.504018461 |
| hsa-miR-331-3p-4373046 | 1.16E-02 | 2.284996009 |
| hsa-miR-338-5P-002658 | 4.49E-02 | 19.10631504 |
| hsa-miR-339-3p-4395295 | 1.42E-02 | 1.783263521 |
| hsa-miR-339-5p-4395368 | 4.57E-06 | 5.431052766 |
| hsa-miR-33a-002135 | 1.22E-02 | -4.878248427 |
| hsa-miR-33a#-002136 | 3.27E-02 | 2.786642187 |
| hsa-miR-342-5p-4395258 | 5.61E-06 | 8.082370398 |
| hsa-miR-346-4373038 | 8.74E-06 | 19.77404292 |
| hsa-miR-34a-4395168 | 5.76E-04 | 10.681118 |
| hsa-miR-34b-002102 | 1.82E-02 | 2.998803041 |
| hsa-miR-361-3p-002116 | 6.85E-03 | 5.23708415 |
| hsa-miR-363-4378090 | 0.043405202 | 2.245197856 |
| hsa-miR-381-4373020 | 1.02E-03 | 5.954411334 |
| hsa-miR-382-4373019 | 2.83E-04 | 10.04245433 |
| hsa-miR-422a-4395408 | 2.99E-02 | -1.857459281 |
| hsa-miR-425-4380926 | 4.44E-02 | 1.873864722 |
| hsa-miR-433-4373205 | 4.58E-05 | 12.24660243 |
| hsa-miR-452-4395440 | 4.27E-02 | -3.709429697 |
| hsa-miR-454#-001996 | 0.018152174 | -2.005389999 |
| hsa-miR-483-3p-002339 | 2.59E-03 | 8.693342372 |
| hsa-miR-483-5p-4395449 | 1.25E-02 | 2.862709767 |
| hsa-miR-490-3p-4373215 | 2.04E-04 | 10.46622322 |
| hsa-miR-491-5p-4381053 | 1.10E-02 | 3.401489337 |
| hsa-miR-500-4395539 | 9.74E-03 | 2.39457988 |
| hsa-miR-501-5p-4373226 | 0.023204132 | 3.239875065 |
| hsa-miR-502-5p-4373227 | 0.018152174 | 2.531136919 |
| hsa-miR-503-4373228 | 7.88E-03 | -5.289241176 |
| hsa-miR-505-4395200 | 3.25E-04 | 9.946869968 |
| hsa-miR-509-5p-4395346 | 1.16E-02 | 6.192205292 |
| hsa-miR-511-4373236 | 1.21E-03 | 7.178292954 |
| hsa-miR-512-3p-4381034 | 8.29E-03 | 5.949514113 |
| hsa-miR-516-3p-001149 | 1.17E-06 | 9.900354362 |
| hsa-miR-518b-4373246 | 2.01E-05 | 14.99065596 |
| hsa-miR-518d-3p-437324[illegible] | 3.67E-03 | 5.471235324 |
| hsa-miR-518e-4395506 | 1.45E-03 | 6.342488808 |
| hsa-miR-519b-3p-002384 | 1.18E-03 | 198.4781478 |
| hsa-miR-519e#-001166 | 1.65E-02 | 20.06928477 |
| hsa-miR-520D-3P-002743 | 3.73E-02 | -5.17E+01 |
| hsa-miR-523-4395497 | 1.25E-02 | -8.741683061 |
| hsa-miR-532-3p-4395466 | 3.88E-02 | 2.037763786 |
| hsa-miR-541#-002200 | 2.67E-03 | 13.55644133 |
| hsa-miR-545#-002266 | 1.16E-02 | -3.397616128 |
| hsa-miR-550-001544 | 7.80E-03 | -2.726772985 |
| hsa-miR-551a-001519 | 6.33E-03 | -4.28E+02 |
| hsa-miR-552-001520 | 5.02E-03 | -5.008521074 |
| hsa-miR-554-001522 | 1.53E-03 | -7.213886654 |
| hsa-miR-564-001531 | 3.06E-03 | 4.729719943 |
| hsa-miR-571-001613 | 5.01E-05 | 26.34572571 |
| hsa-miR-572-001614 | 4.17E-02 | 4.209140668 |
| hsa-miR-579-4395509 | 7.93E-03 | 4.449231119 |

| | | |
|---|---|---|
| hsa-miR-589-001543 | 4.49E-02 | 2.248689528 |
| hsa-miR-590-3P-002677 | 1.12E-06 | -6.148265339 |
| hsa-miR-596-001550 | 3.78E-03 | 3.475990106 |
| hsa-miR-598-4395179 | 3.46E-02 | 2.419220487 |
| hsa-miR-603-001566 | 1.10E-02 | 6.313796875 |
| hsa-miR-605-001568 | 5.54E-08 | 24.96213381 |
| hsa-miR-621-001598 | 9.55E-03 | 14.60683904 |
| hsa-miR-623-001555 | 2.15E-05 | 18.51062794 |
| hsa-miR-624-4395541 | 0.028117499 | -3.149371941 |
| hsa-miR-628-5p-4395544 | 1.85E-02 | -4.076960391 |
| hsa-miR-631-001564 | 1.10E-02 | -7.69456468 |
| hsa-miR-636-4395199 | 1.45E-04 | 4.894690324 |
| hsa-miR-638-001582 | 3.53E-05 | 18.76820952 |
| hsa-miR-639-001583 | 1.45E-02 | 5.352819212 |
| hsa-miR-642-4380995 | 3.53E-05 | 3.956162053 |
| hsa-miR-643-001594 | 5.61E-06 | 21.77515273 |
| hsa-miR-648-001601 | 1.64E-02 | 5.845054219 |
| hsa-miR-649-001602 | 2.85E-03 | -9.87249353 |
| hsa-miR-650-001603 | 1.85E-02 | 3.055916306 |
| hsa-miR-652-4395463 | 5.76E-04 | 4.913369207 |
| hsa-miR-658-001513 | 6.33E-03 | 170076.9646 |
| hsa-miR-659-001514 | 5.61E-06 | 29.89586624 |
| hsa-miR-660-4380925 | 2.33E-02 | 2.834791775 |
| hsa-miR-661-001606 | 1.95E-02 | 3.952455757 |
| hsa-miR-668-001992 | 3.46E-02 | -4.129300766 |
| hsa-miR-672-4395438 | 8.02E-07 | 17.79716177 |
| hsa-miR-720-002895 | 3.45E-04 | -2.997126757 |
| hsa-miR-769-5p-001998 | 2.42E-02 | 2.728704422 |
| hsa-miR-770-5p-002002 | 2.58E-02 | 4.598746269 |
| hsa-miR-9-4373285 | 1.74E-04 | 7.411103918 |
| hsa-miR-922-002152 | 5.98E-05 | -5.132132548 |
| hsa-miR-92a-4395169 | 2.89E-02 | 2.304960917 |
| hsa-miR-93-4373302 | 3.46E-02 | 2.616279329 |
| hsa-miR-935-002178 | 1.35E-05 | -1.31E+01 |
| hsa-miR-939-002182 | 1.29E-03 | 5.369590891 |
| hsa-miR-95-4373011 | 1.10E-02 | 2.713101175 |
