# Supplementary figures and images for: MicroRNA regulation and its effects on cellular transcriptome in Human Immunodeficiency Virus-1 (HIV-1) infected individuals with distinct viral load and CD4 cell counts
Source: BMC Infect Dis. 2013 May 30;13:250. doi: 10.1186/1471-2334-13-250 (PMC3680326; doi:10.1186/1471-2334-13-250)

Figure S1

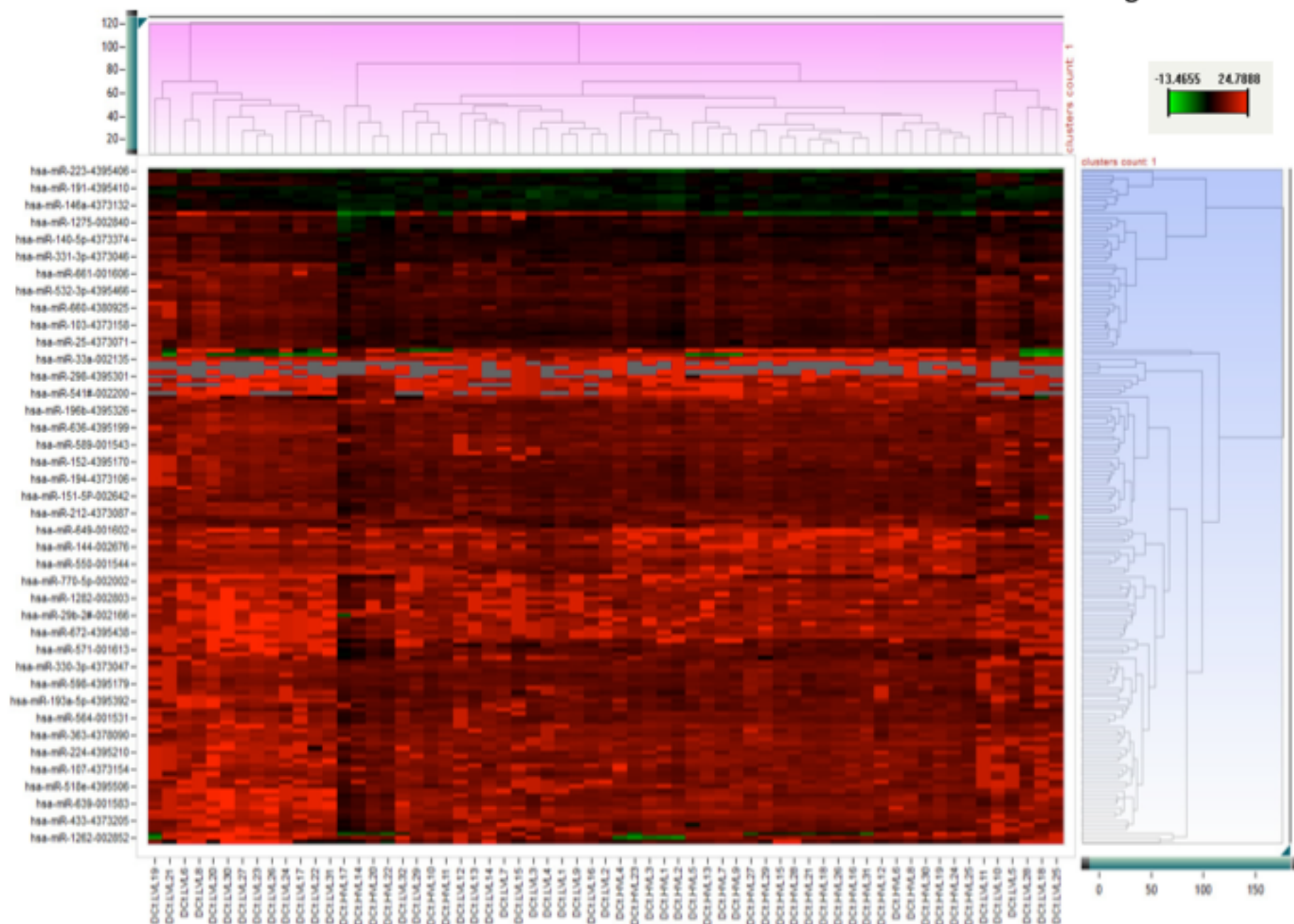

Supplement: Additional file 2: Figure S1 — Hierarchical clustering of miRNAs in HIV-1 infected low viral load (LVL) versus high viral load (HVL) subjects. The dendogram depicting the clustering of samples is calculated using Complete linkage with Euclidian distance measure values. Color ranging from green to red indicates minimum to maximum dCT. Numbers on Xaxis represent subject group. The clustergrams were generated using data from StatMiner analyses. [file 1471-2334-13-250-S2.pdf]

Figure S2

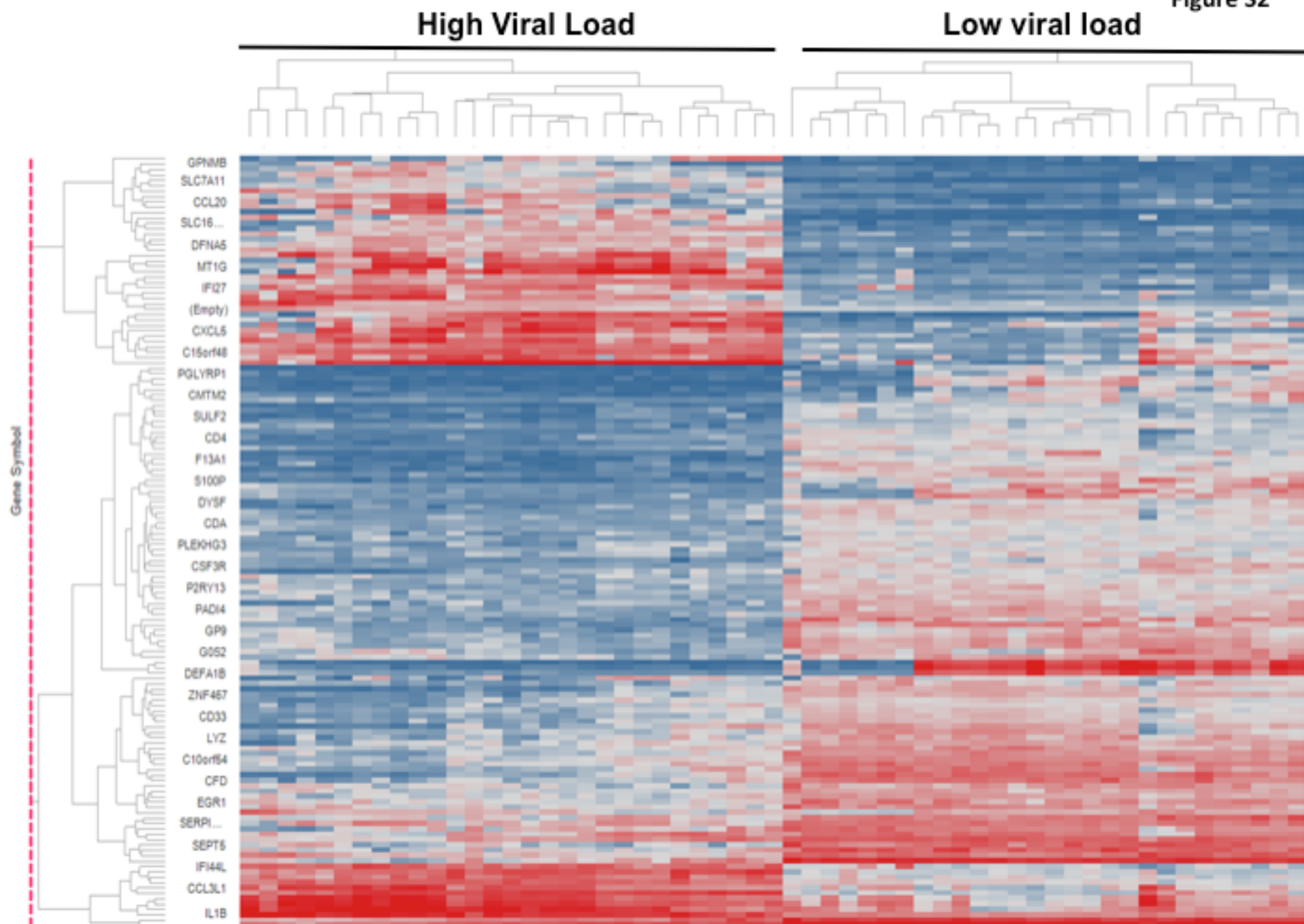

Supplement: Additional file 4: Figure S2 — Hierarchical clustering of differentially regulated mRNA in low viral load (LVL) ompared to high viral load (HVL) subjects. The probes in this clustergram are significantly differentially regulated (p < 0.05). Red indicates high, blue indicates low, and gray stands for no change in level of expression. Numbers on X-axis represent subject group; Y-axis represents the gene symbol. [file 1471-2334-13-250-S4.pdf]

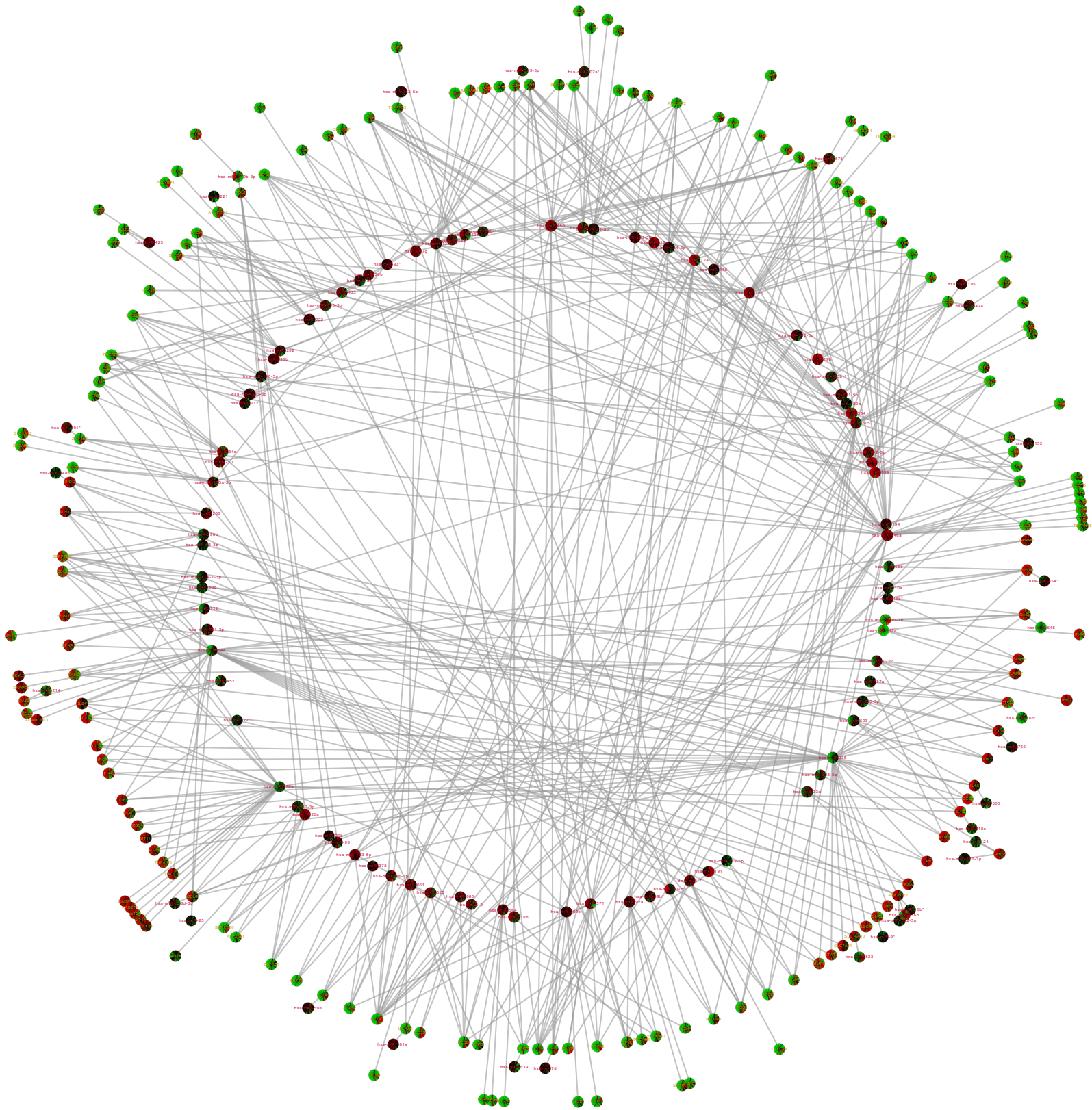

Supplement: Additional file 6: Figure S3 — Network of predicted miRNA-mRNA interactions by GroupMiR using results from miRNA and mRNA array and visualized by Cytoscape. Regression-based method was used to predict the potential miRNA (circles) that actively regulate mRNA (squares). Differentially regulated miRNA and mRNA in HIV-1 infected subjects compared to the uninfected controls were used to predict the miRNA-mRNA pairs. Red and green within the mRNA represents up and down regulation, respectively. Each slice within the circle represents HIV-1 infected subject. [file 1471-2334-13-250-S6.pdf]
