# Supplementary material for: MicroRNA regulation and its effects on cellular transcriptome in Human Immunodeficiency Virus-1 (HIV-1) infected individuals with distinct viral load and CD4 cell counts
Source: BMC Infect Dis. 2013 May 30;13:250. doi: 10.1186/1471-2334-13-250 (PMC3680326; doi:10.1186/1471-2334-13-250)
Supplement: Additional file 3: Table S2 — Differentially regulated mRNA probes in low (LVL) and high viral load (HVL) groups compared to uninfected control group with statistical significance. [file 1471-2334-13-250-S3.pdf]

Table S2: Differentially regulated mRNA probes in low (LVL) and high viral load (HVL) groups compared to uninfected control group with statistical significance.

Control vs LVL

Gene	Fold change	p value
FASTK	-0.31	0.0075
RNF165	-0.62	0.0159
CD8B	0.84	0.0159
APOBEC3H	0.76	0.0159
RCAN1	0.32	0.0159
TNFRSF9	0.45	0.0159
APOBEC3G	0.81	0.0182
CDCA7	0.49	0.0198
PCGF6	0.43	0.0210
KRT73	-0.72	0.0210
MAPK14	0.48	0.0210
CD8A	0.95	0.0210
COLQ	-0.65	0.0281
HDGFRP3	0.52	0.0295
HGD	-0.57	0.0316
LAG3	1.03	0.0316
LTB4R	-0.50	0.0346
REEP3	0.35	0.0346
UEVLD	0.48	0.0346
HDLBP	0.34	0.0368
MLST8	-0.36	0.0371
PPP2R3C	0.31	0.0372
B3GNT2	0.48	0.0386
GCLM	0.34	0.0416
IL12A	0.30	0.0416
TMEM110	0.33	0.0416
GALK2	0.44	0.0416
COLQ	-0.49	0.0426
RAB28	0.47	0.0426
APOL6	0.58	0.0426
SCD	0.61	0.0427
UBE2G1	0.43	0.0427
NIPA2	0.29	0.0442
KIAA1671	0.56	0.0442
RAB27A	0.50	0.0442
CCL4L2	0.83	0.0442
RPL29	0.44	0.0442
PPP2R5C	0.63	0.0442
SERPINB9	0.49	0.0442
DDX28	-0.26	0.0471
PDE5A	-0.77	0.0471

Control vs HVL

Gene Symbol	Fold change	p value
CSF3R	-4.47	3.08917E-31
FCER1A	-4.70	6.2839E-30
NRGN	-4.03	1.69859E-29
SC4MOL	2.03	1.43024E-28
CD1D	-3.86	1.10664E-27
TUBB1	-4.00	1.15886E-27
F13A1	-3.90	1.64447E-27
LAMP3	3.04	1.84997E-27
SEPT5	-4.40	3.55374E-27
ZNF185	-2.35	5.24159E-27
PRAM1	-3.29	6.7846E-27
RBP7	-2.34	6.7846E-27
PADI4	-3.41	8.33556E-27
SULF2	-2.18	2.53951E-26
GP9	-3.43	2.7448E-26
CCL22	3.46	3.41742E-26
SLC40A1	-3.00	5.1597E-26
ABL1	-2.24	5.87983E-26
KLF4	-3.21	6.53253E-26
NFE2	-3.88	6.53253E-26
TSPAN18	-2.51	1.5286E-25
CD4	-2.77	2.99654E-25
LOC255809	-2.27	2.99654E-25
ASGR2	-3.29	6.481E-25
GPBAR1	-3.19	7.84631E-25
SDPR	-3.54	9.46121E-25
CDA	-2.52	3.5875E-24
FCN1	-4.84	4.11135E-24
ASGR2	-2.88	4.22835E-24
SULF2	-3.15	6.82333E-24
RN7SK	2.59	7.79154E-24
VENTX	-2.48	8.99069E-24
CDH23	-2.29	9.14248E-24
INDO	5.18	1.4433E-23
C22orf32	-2.26	3.75003E-23
CLK1	2.31	7.04953E-23
CREB5	-2.08	8.29337E-23
GPR68	2.69	9.42092E-23
TBC1D2B	2.33	1.26722E-22
IRS2	-2.12	1.55104E-22
SH3BGRL2	-2.20	1.57529E-22

LVL vs HLV

Gene Symbol	Fold change	p value
RN7SK	2.55	5.74963E-19
CPVL	-4.61	1.08185E-15
LILRA5	-2.45	3.14747E-15
MGC33556	-2.04	3.14747E-15
ASGR2	-2.83	1.03371E-14
ZNF467	-3.23	1.32902E-14
GPBAR1	-3.07	1.42469E-14
P2RY13	-2.48	2.43284E-14
KBTBD11	-2.37	2.58391E-14
FCN1	-4.43	4.73849E-14
CRISPLD2	-2.28	6.31281E-14
LST1	-3.61	7.06224E-14
CD4	-2.33	9.37294E-14
CD1D	-3.47	1.08847E-13
CSF3R	-3.56	1.35361E-13
ASGR1	-3.46	1.56621E-13
PLEKHG3	-2.25	1.59962E-13
KLF4	-3.04	1.90506E-13
S100A12	-2.91	1.972E-13
C10orf54	-3.09	2.32545E-13
SLC40A1	-2.33	2.64164E-13
LILRA5	-2.25	3.06848E-13
F13A1	-3.19	3.48486E-13
SULF2	-2.60	3.80433E-13
ALDH2	-2.60	6.6544E-13
SIGLEC10	-2.96	6.91658E-13
LST1	-2.81	7.32989E-13
FPR1	-3.02	8.08659E-13
FLJ22662	-3.23	8.43339E-13
ASGR2	-2.25	9.40448E-13
PRAM1	-2.68	1.10027E-12
MS4A6A	-3.71	1.38325E-12
PTGR1	2.11	3.10083E-12
NFE2	-3.25	3.60162E-12
CDA	-2.23	4.83786E-12
UBE2L3	-2.64	5.16536E-12
LOC255809	-2.08	5.42148E-12
NRGN	-3.06	5.42148E-12
HSPA1B	2.33	6.78208E-12
GARNL4	-2.19	8.15183E-12
CD33	-2.54	8.46584E-12

FOSB	-4.20	2.54795E-22
MS4A6A	-4.15	2.54795E-22
FASLG	2.31	4.57399E-22
H3F3B	-2.32	4.57399E-22
FLJ22662	-3.56	4.67931E-22
CEBPD	-2.54	4.75611E-22
SLC7A11	3.21	5.38607E-22
CD302	-2.95	6.26405E-22
NCR3	-2.30	8.18219E-22
SIGLEC10	-3.33	8.64867E-22
HSPA1B	2.91	9.25685E-22
ADAM15	-2.17	9.81688E-22
CPVL	-4.91	1.15484E-21
UBE2L3	-2.89	1.18986E-21
VIPR1	-2.03	1.31218E-21
ZNF467	-3.60	1.59469E-21
CSF3R	-2.79	3.14563E-21
ACRBP	-2.83	3.67724E-21
MTMR11	-2.71	3.69239E-21
TXN	2.33	3.85281E-21
CRIP2	-2.39	3.92003E-21
IFIH1	2.08	4.00766E-21
EGR1	-2.71	4.35058E-21
IFI27	5.17	4.60582E-21
KBTBD11	-2.76	6.47045E-21
TESC	-2.44	6.60751E-21
ADAMDEC1	4.07	6.87542E-21
LY86	-2.53	8.47364E-21
PTCRA	-2.56	1.48972E-20
LOC338758	-2.56	2.58937E-20
GARNL4	-2.55	2.63198E-20
MGC33556	-2.32	2.80479E-20
RSAD2	3.34	4.20905E-20
DYSF	-2.80	6.58576E-20
IMPA2	-2.72	7.50398E-20
MMP25	-2.69	7.55962E-20
P2RY13	-2.60	1.05184E-19
C10orf54	-3.49	1.25172E-19
DFNA5	2.76	1.25695E-19
TCEA3	-2.45	1.3157E-19
PELI2	-2.36	1.51885E-19
ESAM	-2.14	1.71834E-19
BCL11A	-2.01	1.98487E-19
NPC1	2.03	1.98487E-19
IFI44	2.26	2.08457E-19
SERPINB2	4.42	2.20944E-19
FOLR3	-3.36	2.70063E-19

LYZ	-2.95	1.10873E-11
PADI4	-2.82	1.23022E-11
DYSF	-2.49	1.31783E-11
PTCRA	-2.06	1.55681E-11
FCER1A	-3.71	1.55691E-11
TESC	-2.19	1.57962E-11
MS4A6A	-3.51	2.16497E-11
CD302	-2.53	2.59293E-11
SERPINA1	-2.15	2.59293E-11
CFD	-3.55	3.4223E-11
GPBAR1	-2.03	3.78848E-11
AIF1	-2.53	4.18679E-11
CSF3R	-2.17	4.4846E-11
S100P	-3.41	5.83731E-11
LILRA2	-2.59	7.6603E-11
SDPR	-2.51	8.07423E-11
MTMR11	-2.40	8.52348E-11
IFI27	4.12	9.48695E-11
TUBB1	-2.97	1.26546E-10
SLC7A11	2.75	1.36916E-10
AIF1	-2.59	1.38315E-10
FOSB	-3.56	1.39319E-10
RNASE2	-2.77	1.54899E-10
S100A8	-2.06	2.05669E-10
GP9	-2.63	2.15243E-10
LGALS2	-2.51	2.33067E-10
CD300LF	-2.19	2.38995E-10
FOLR3	-2.97	2.796E-10
ACRBP	-2.07	3.35451E-10
SEPT5	-3.37	4.37179E-10
FOS	-3.34	4.56405E-10
CST3	-2.39	7.6249E-10
SERPINB2	3.66	7.66078E-10
SERPINB2	3.96	8.28068E-10
KYNU	2.11	9.32262E-10
CLEC7A	-2.48	9.43527E-10
LY86	-2.07	1.05078E-09
KLF11	-2.11	1.18605E-09
RSAD2	2.78	1.70691E-09
MNDA	-2.40	1.71484E-09
KLF10	-2.02	1.92444E-09
DFNA5	2.25	2.63572E-09
MS4A6A	-2.98	2.86333E-09
ALDH1A1	-2.45	3.71077E-09
CLEC7A	-2.35	3.85728E-09
IL1A	3.39	4.16978E-09
EGR1	-2.18	4.51329E-09

PTGDS	-2.22	2.71536E-19
LST1	-4.00	2.82377E-19
SERPINB2	4.76	3.0353E-19
ASGR1	-3.81	3.89103E-19
CAMP	-3.82	4.19281E-19
LST1	-3.16	4.82681E-19
TOP1MT	-2.13	5.12317E-19
NLRP12	-2.04	5.26186E-19
NACC2	-2.24	6.29934E-19
RNASE2	-2.80	7.62484E-19
CTTN	-2.31	8.21113E-19
ALDH1A1	-2.11	9.11421E-19
TMEM91	-2.19	1.03396E-18
PTGS1	-2.53	1.15197E-18
CFD	-4.08	1.18566E-18
EPSTI1	2.81	1.20529E-18
LILRA5	-2.45	1.39565E-18
GNG11	-2.84	1.45371E-18
ZNF101	-2.27	2.12786E-18
MGC13057	-2.33	2.30681E-18
APOBEC3G	2.03	3.02425E-18
RGL1	3.38	3.02425E-18
ALDH2	-2.92	3.2747E-18
ADAMDEC1	3.01	5.10843E-18
GBP4	2.03	5.21671E-18
S100P	-3.86	5.31152E-18
C15orf48	3.36	5.80753E-18
KYNU	2.67	6.24512E-18
APOBEC3G	2.06	8.24566E-18
OAS2	2.31	8.34597E-18
PLA2G7	3.62	9.91615E-18
GPBAR1	-2.04	1.02265E-17
IFI44L	3.51	1.08176E-17
C15orf48	4.55	1.33969E-17
FPR1	-3.32	1.48722E-17
CRISPLD2	-2.34	1.61757E-17
HDC	-2.68	1.78142E-17
ETNK1	-2.21	1.79279E-17
FLJ14213	2.24	2.05628E-17
LILRA5	-2.53	2.23675E-17
HSPA1A	2.13	2.3059E-17
IL1A	3.95	2.73317E-17
RRP12	-2.42	3.01283E-17
UBXN11	-2.01	3.0378E-17
LCN2	-2.22	3.17245E-17
ALDH1A1	-2.60	3.42898E-17
TIGIT	2.06	4.58636E-17
SERPINA1	-2.16	5.03785E-09
TXN	2.01	5.31189E-09
TSPAN32	-2.10	5.85454E-09
LOC338758	-2.04	1.07979E-08
MGST2	-2.00	1.11041E-08
GAS7	-2.04	1.11112E-08
IMPA2	-2.17	1.16E-08
ALDH1A1	-2.04	1.4097E-08
RNASE3	-2.26	1.61852E-08
CXCL5	4.04	1.72056E-08
CAMP	-3.34	1.77376E-08
IFI44L	2.66	1.94135E-08
CXCL2	2.70	2.24289E-08
CXCL5	4.07	2.42701E-08
ADAMDEC1	3.16	3.26322E-08
FCGR3B	-2.84	3.51925E-08
TNIP3	2.05	3.84323E-08
GPR68	2.01	3.97374E-08
C15orf48	3.87	4.49847E-08
GNG11	-2.08	4.75031E-08
CLEC12A	-2.64	4.94018E-08
PLA2G7	2.84	5.01621E-08
IL24	2.63	6.21772E-08
LAMP3	2.38	6.62223E-08
CYP1B1	2.14	8.12779E-08
CTSL1	3.20	9.17511E-08
DEFA3	-5.12	9.6738E-08
ADAMDEC1	2.39	9.90247E-08
DEFA4	-2.86	1.11436E-07
FGL2	-2.24	1.16369E-07
DEFA1	-5.13	1.27624E-07
CCL3	2.42	1.38752E-07
C15orf48	2.75	1.41724E-07
CTSL1	3.01	1.45093E-07
CCL3L3	2.47	1.53625E-07
IL6	3.28	1.71139E-07
CCL3L1	2.42	1.73813E-07
IL19	2.17	1.79719E-07
CCL20	2.68	2.05136E-07
KYNU	2.07	2.38878E-07
CD36	-2.85	2.43704E-07
TNFAIP6	2.45	2.48691E-07
DEFA1B	-4.07	2.99217E-07
DEFA1B	-3.96	3.23549E-07
CLC	-2.91	3.52076E-07
CD36	-2.64	4.16825E-07
IL19	2.11	5.37336E-07

LGALS2	-2.79	4.65195E-17
CACNA2D3	-2.04	8.29607E-17
CTSL1	3.74	8.39503E-17
PTGR1	2.26	1.0145E-16
S100A4	-2.10	1.07747E-16
PECAM1	-2.09	1.2719E-16
KLF11	-2.55	1.41761E-16
CTSL1	3.98	1.41782E-16
PMP22	2.65	1.69748E-16
GBP1	2.68	1.75688E-16
MT1G	4.93	2.00856E-16
GRASP	-2.24	2.19423E-16
DEFA1	-5.60	2.22304E-16
S100A12	-2.90	2.24348E-16
OAF	-2.06	2.5316E-16
CD33	-2.64	2.56184E-16
MPL	-2.35	2.64101E-16
C1orf162	-2.13	3.20636E-16
TSPAN9	-2.33	3.78347E-16
CCL3	3.36	4.11467E-16
SLC39A8	2.40	4.54052E-16
EOMES	2.53	5.22693E-16
FOS	-3.77	5.27815E-16
GK	2.22	5.27815E-16
CCL4L2	2.55	6.23611E-16
MS4A6A	-3.19	6.52902E-16
PLSCR1	2.21	7.1144E-16
ACTA2	2.12	7.45184E-16
MS4A6A	-3.69	7.80237E-16
TMEM154	-2.24	8.82659E-16
WWP2	-2.22	8.83608E-16
TNIP3	2.49	9.71886E-16
CXCL5	4.53	1.11414E-15
PLEKHG3	-2.33	1.33664E-15
TSPAN32	-2.58	1.33664E-15
VCAM1	2.01	1.82089E-15
BST1	-2.08	2.28021E-15
GK	2.28	2.43187E-15
TXN	2.59	3.56663E-15
GBP1	2.50	3.70877E-15
CCL8	5.11	4.31348E-15
MAL	-2.48	4.71171E-15
ITGAM	-2.20	4.81262E-15
IL4I1	2.45	5.51817E-15
CXCL5	4.58	6.3312E-15
CCL3L1	3.16	7.48114E-15
CMTM2	-3.39	8.79261E-15

CCL22	2.67	6.57089E-07
CMTM2	-2.56	6.97104E-07
FCGR1B	-2.22	7.03686E-07
INDO	3.85	8.13923E-07
LOC653600	-3.00	9.45288E-07
EMP1	2.38	1.01688E-06
CCL3L1	2.49	1.29932E-06
IL8RB	-2.01	1.42228E-06
CLEC12A	-2.01	1.42247E-06
RGL1	2.57	1.46892E-06
PMP22	2.05	1.57314E-06
MT1G	3.87	1.77544E-06
PGLYRP1	-2.02	1.80649E-06
VNN2	-2.25	3.0958E-06
EBI3	2.11	3.18909E-06
IL1B	2.47	4.19842E-06
CTSL1	2.75	4.20813E-06
PI3	-2.10	4.84609E-06
OLR1	3.00	6.66138E-06
G0S2	-2.12	9.90973E-06
CCL2	3.95	1.4194E-05
VCAN	-2.19	1.65714E-05
MT1H	3.29	2.14549E-05
THBS1	2.50	2.57742E-05
CCL8	3.89	5.18302E-05
PMP22	2.51	7.0973E-05
SLC16A10	2.03	7.49933E-05
MT1M	2.61	0.000161575
GPNMB	2.03	0.00068177
CCL7	2.80	0.002497504
MMP9	2.12	0.005380915

MGST2	-2.33	1.12634E-14
MS4A3	-2.13	1.21241E-14
STAT1	2.15	1.40578E-14
MT2A	2.34	1.4466E-14
KYNU	2.62	1.58792E-14
HK3	-2.10	1.77897E-14
CLEC1B	-2.49	1.93053E-14
FCGR3B	-3.51	2.16455E-14
IL6	4.01	2.2412E-14
LYZ	-3.07	2.33199E-14
CD8A	2.03	2.44667E-14
CTSL1	3.62	2.5176E-14
RASGRP2	-2.04	3.43329E-14
MT1H	4.39	4.26585E-14
EBI3	2.66	4.44194E-14
FCER2	-2.03	4.66219E-14
DEFA3	-5.28	5.87893E-14
S100A8	-2.20	6.05402E-14
CST3	-2.62	6.91289E-14
CCL3L3	2.86	8.82671E-14
OASL	2.27	1.05933E-13
CLEC7A	-2.82	1.1735E-13
DEFA1B	-4.06	1.19909E-13
CD300LF	-2.37	1.81105E-13
GAS7	-2.46	3.10926E-13
ANKRD22	2.58	5.36664E-13
CLC	-3.76	6.17418E-13
DEFA1B	-3.97	6.83043E-13
EMR3	-2.03	1.09145E-12
LILRA2	-2.71	1.48517E-12
ISG15	2.07	1.55015E-12
LAG3	2.29	1.76293E-12
OLR1	3.80	2.00409E-12
TNFAIP6	2.89	2.03457E-12
CLEC12A	-2.22	2.22845E-12
CXCL2	2.97	2.98962E-12
CLEC7A	-2.65	3.38696E-12
PYGL	-2.01	3.4907E-12
CXCL10	3.22	3.70783E-12
IL8RB	-2.61	3.70808E-12
TMEM51	2.26	5.10541E-12
CCL3L1	3.06	5.1372E-12
PGLYRP1	-2.24	5.84565E-12
CYP1B1	2.47	7.41646E-12
AIF1	-2.67	7.62445E-12
MT1A	2.39	7.66469E-12
PMP22	3.27	1.01184E-11

TREM1	-2.42	1.17539E-11
MT1E	2.73	1.97499E-11
MTE	2.21	2.23045E-11
IL1B	3.11	2.83655E-11
AIF1	-2.61	3.23728E-11
CTSS	-2.07	4.06676E-11
CD36	-3.08	4.08456E-11
SELL	-2.06	4.62691E-11
CLEC12A	-2.55	5.52244E-11
CCL2	4.53	6.47935E-11
SLC16A10	2.45	7.83853E-11
RGS18	-2.52	8.94012E-11
EMP1	2.62	1.08689E-10
SERPINA1	-2.23	1.14818E-10
MT1M	3.36	1.22356E-10
G0S2	-2.38	1.29387E-10
KIAA1199	2.74	1.44367E-10
TCN1	-2.23	2.06589E-10
IL19	2.36	2.73108E-10
CCL20	2.97	2.89921E-10
KRT72	-2.10	4.2269E-10
MME	-2.37	4.34231E-10
MNDA	-2.31	5.05325E-10
CD36	-2.78	6.56495E-10
IL24	2.72	7.87686E-10
MT1X	2.01	1.90526E-09
VCAN	-2.70	2.73249E-09
IL19	2.26	3.32635E-09
CCL24	2.19	3.88096E-09
PI3	-2.57	3.90308E-09
FGL2	-2.21	5.66177E-09
LOC653600	-2.51	7.42818E-09
GPNMB	2.79	1.01482E-08
DEFA4	-2.29	1.76729E-08
IFIT1	2.07	2.03323E-08
PROK2	-2.05	5.2929E-08
ALAS2	-2.34	5.66718E-08
LOC730249	2.22	5.67352E-08
IFNG	2.73	6.28227E-08
FPR3	2.67	7.83368E-08
SLC39A8	2.11	1.97899E-07
CCL3L1	2.22	1.98038E-07
CCL7	3.61	3.00985E-07
CCL13	2.27	3.68047E-07
SERPINA1	-2.05	4.39056E-07
MMP9	2.96	1.00968E-06
IL8	2.00	1.07607E-06

CD93	-2.03	1.4849E-06
THBS1	2.29	2.20276E-06
VNN2	-2.20	4.24463E-06
RNASE1	2.09	4.49623E-05
