# Supplementary material for: MicroRNA regulation and its effects on cellular transcriptome in Human Immunodeficiency Virus-1 (HIV-1) infected individuals with distinct viral load and CD4 cell counts
Source: BMC Infect Dis. 2013 May 30;13:250. doi: 10.1186/1471-2334-13-250 (PMC3680326; doi:10.1186/1471-2334-13-250)
Supplement: Additional file 5: Table S3 — a: Top ten Canonical pathways representing mRNA that are differentially expressed in LVL group compared to uninfected seronegative subjects. b: Top ten Canonical pathways representing mRNA that are differentially expressed in HVL group compared to uninfected seronegative subjects. [file 1471-2334-13-250-S5.docx]

|  |  |  |  |
| --- | --- | --- | --- |
|  |  |  |  |

**Table S3a:** Top ten Canonical pathways representing mRNA that are differentially expressed in LVL group compared to uninfected seronegative subjects.

| **Canonical Pathways** | **-log(p-value)** | **Ratio** |
| --- | --- | --- |
| Protein Kinase A Signaling | 2.35E-01 | 2.69E-03 |
| Molecular Mechanisms of Cancer | 2.59E-01 | 2.76E-03 |
| Role of Macrophages, Fibroblasts and Endothelial Cells in Rheumatoid Arthritis | 3.04E-01 | 9.9E-03 |
| Glucocorticoid Receptor Signaling | 3.38E-01 | 9.8E-03 |
| Protein Ubiquitination Pathway | 3.43E-01 | 9.09E-03 |
| Cardiac Hypertrophy Signaling | 3.92E-01 | 1.01E-02 |
| Role of Osteoblasts, Osteoclasts and Chondrocytes in Rheumatoid Arthritis | 3.96E-01 | 1.01E-02 |
| Breast Cancer Regulation by Stathmin1 | 4.40E-01 | 1.02E-02 |
| Leukocyte Extravasation Signaling | 4.42E-01 | 1.05E-02 |
| Thrombin Signaling | 4.46E-01 | 1.03E-02 |

**Table S3b:** Top ten Canonical pathways representing mRNA that are differentially expressed in HVL group compared to uninfected seronegative subjects.

| **Canonical Pathways** | **-log(p-value)** | **Ratio** |
| --- | --- | --- |
| Antiproliferative Role of Somatostatin Receptor 2 | 2.08E-01 | 9.92E-02 |
| GM-CSF Signaling | 2.08E-01 | 1.95E-01 |
| Role of PI3K/AKT Signaling in the Pathogenesis of Influenza | 2.12E-01 | 1.86E-01 |
| Role of BRCA1 in DNA Damage Response | 2.17E-01 | 1.08E-01 |
| Induction of Apoptosis by HIV1 | 2.21E-01 | 1.79E-01 |
| Retinoic acid Mediated Apoptosis Signaling | 2.21E-01 | 2.61E-01 |
| Cell Cycle: G1/S Checkpoint Regulation | 2.21E-01 | 8.57E-02 |
| Calcium-induced T Lymphocyte Apoptosis | 2.26E-01 | 7.94E-02 |
| Myc Mediated Apoptosis Signaling | 2.26E-01 | 1.23E-01 |
| Death Receptor Signaling | 2.26E-01 | 3.08E-01 |
